# 5-HT_3_ Receptors on Mitochondria Influence Mitochondrial Function

**DOI:** 10.3390/ijms24098301

**Published:** 2023-05-05

**Authors:** Santosh T. R. B. Rao, Ilona Turek, Julian Ratcliffe, Simone Beckham, Cassandra Cianciarulo, Siti S. B. M. Y. Adil, Christine Kettle, Donna R. Whelan, Helen R. Irving

**Affiliations:** 1 La Trobe Institute for Molecular Science, La Trobe University, P.O. Box 199, Bendigo, VIC 3552, Australia; s.tata@latrobe.edu.au (S.T.R.B.R.);; 2 Department of Rural Clinical Sciences, La Trobe University, P.O. Box 199, Bendigo, VIC 3552, Australia; 3 Bio Imaging Platform, La Trobe University, Kingsbury Dr, Bundoora, VIC 3086, Australia; 4 Regional Science Operations, La Trobe University, P.O. Box 199, Bendigo, VIC 3552, Australia

**Keywords:** 5-hydroxytryptamine 3 (5-HT_3_) receptor, ion channel subunits, ligand-gated ion channels, mitochondria, ondansetron, immunogold staining, serotonin type 3 receptor

## Abstract

The 5-hydroxytryptamine 3 (5-HT_3_) receptor belongs to the pentameric ligand-gated cation channel superfamily. Humans have five different 5-HT_3_ receptor subunits: A to E. The 5-HT_3_ receptors are located on the cell membrane, but a previous study suggested that mitochondria could also contain A subunits. In this article, we explored the distribution of 5-HT_3_ receptor subunits in intracellular and cell-free mitochondria. Organelle prediction software supported the localization of the A and E subunits on the inner membrane of the mitochondria. We transiently transfected HEK293T cells that do not natively express the 5-HT_3_ receptor with an epitope and fluorescent protein-tagged 5HT3A and 5HT3E subunits. Fluorescence microscopy and cell fractionation indicated that both subunits, A and E, localized to the mitochondria, while transmission electron microscopy revealed the location of the subunits on the mitochondrial inner membrane, where they could form heteromeric complexes. Cell-free mitochondria isolated from cell culture media colocalized with the fluorescent signal for A subunits. The presence of A and E subunits influenced changes in the membrane potential and mitochondrial oxygen consumption rates upon exposure to serotonin; this was inhibited by pre-treatment with ondansetron. Therefore, it is likely that the 5-HT_3_ receptors present on mitochondria directly impact mitochondrial function and that this may have therapeutic implications.

## 1. Introduction

The 5-hydroxytryptamine (5-HT_3_) receptor is a cysteine loop ligand-gated ion channel belonging to the serotonin receptor family [1]. There are five receptor subunits that can form homomeric (all A subunits) or heteromeric (mixtures of A and either B, C, D, or E subunits) receptors in humans [2,3,4]. The structure of the mouse 5HT3A subunit, which is 84% homologous to the human A subunit [5], has been revealed by X-ray crystallography [6] and cryo-electron microscopy studies [7]. Cryo-electron microscopy studies have revealed ligand-bound conformations of 5-HT_3_A homomeric receptors with the antagonists granisetron, tropisetron, and palonosetron or the natural agonist serotonin [7,8,9,10,11].

5-HT_3_ receptors are expressed in the nervous system and digestive system, where they form targets for 5-HT_3_ receptor antagonists [12,13,14,15]. The human 5-HT_3_ receptor subunits A, B, C, D, and E are expressed differentially in various tissues. For instance, the 5HT3A, 5HT3B, and 5HT3C subunits are highly expressed in different regions of the brain [2,3]. The 5HT3A and 5HT3B subunits are co-expressed in the human spleen, intestine, and some brain regions, such as the amygdala, telencephalon, and entorhinal cortex [16,17]. In humans, the A, B, C and E subunits are differentially expressed throughout the colon and ileum, where the E subunit in particular is expressed at high levels in the mucosal layers of the gut but not the muscular layers [18,19,20].

The translation of the 5-HT_3_ receptor and its trafficking (plasma membrane targeting, and ligand-induced endocytosis) have been studied in live cells by transfecting them with recombinant 5-HT_3_ receptor subunit fusion constructs with fluorescent proteins inserted into the intracellular loop between the transmembrane (TM) 3 and TM 4 domains of the subunit [21,22,23,24]. High cell-surface expression levels of the homopentameric complexes of recombinant 5HT3A subunits occur in transfected human embryonic kidney (HEK293) cells, and these recombinant receptors share considerable pharmacological and functional properties with native neuronal 5-HT_3_ receptors [23,24,25,26,27]. The 5HT3A subunit plays an important role in the expression and localization of other subunits [15]. For example, the 5HT3B subunit is retained in the endoplasmic reticulum (ER) when expressed in the absence of the A subunit; however, the 5HT3B subunit displays cell-surface localization when co-expressed with the 5HT3A subunit [28]. Förster resonance energy transfer (FRET) studies using isolated plasma membrane sheets containing 5-HT_3_ receptors with fluorescent fusion proteins show that AB subunit heteromers assemble in the A3B2 stoichiometry [23]. In addition to the B subunit, the C, D and E subunits modify channel electrical properties when expressed with the 5HT3A subunit [3,29,30,31,32] underscoring their ability to form functional receptor heteromers.

Most interest has focused on the ability of 5-HT_3_ receptors to reach the plasma membrane, where they form a target, particularly for 5-HT_3_ receptor antagonists. At the membrane, they are often found at actin-rich membrane domains such as cell–cell interfaces, signifying the important role of microtubules in 5-HT_3_ receptor localization [22,33,34]. Generally, intracellular 5-HT_3_ receptor subunits have been mainly described in the endoplasmic reticulum [22,28,35]. However, one study reported 5-HT_3_ receptors localizing on mitochondria in mouse cardiomyocytes [36]. During hypoxic conditions, the mitochondria-localized 5-HT_3_ receptors increase Ca^2+^ uptake [36]. However, it is unclear how 5-HT_3_ receptors can reach mitochondrial membranes and their specific location therein. Our in silico predictions suggested that human 5HT3A and 5HT3E subunits contain mitochondrial signal sequences. To visualize the 5HT3A and 5HT3E subunit localization intracellularly, we transfected HEK293T cells with fluorescently tagged 5HT3A and 5HT3E subunits and identified that they co-localized with mitochondria. Further immunogold staining and transmission electron microscopy revealed that these subunits occur on the mitochondrial inner membrane. We also demonstrated that their presence in the mitochondrial inner membrane modulated the mitochondrial membrane’s potential and oxygen consumption when treated with the ligands, serotonin, or ondansetron.

## 2. Results

### 2.1. 5-HT_3_ Receptor Subunit Protein Localization Signal Predictions

In view of the report that mouse cardiomyocytes contained 5-HT_3_ receptors [36], we queried prediction software to determine if any organelle-targeting signals were present in the protein sequences of the different human 5-HT_3_ receptor subunits. SignalP-5.0 and Eukaryotic Linear Motif (ELM) software [37,38] predicted the presence of signal peptides in all 5-HT_3_ receptor subunits (Table 1). Mitochondrial-targeting signals for 5-HT_3_ receptor subunits were predicted using MitoFates and TPpred2 software, which are specific for mitochondrial targeting peptides [39,40]. TPpred2 software predicted that a 43-amino-acid-long N-terminal mitochondrial localization signal was present in the 5HT3E subunit, while no mitochondrial localization signal was detected in the amino acid sequences of the other subunits (Table 1 and Supplementary Figure S1). MitoFates predicted that the 5HT3A subunit had a translocase of the outer mitochondrial membrane complex subunit 20 (TOM20) recognition motif specific to mitochondria between 2 and 6 amino acids at the N-terminal region of the protein sequence. It also predicted that subunit B contained a TOM20 recognition motif specific to mitochondria between 64 and 68 amino acids (Table 1 and Supplementary Figure S2). However, the TOM20 recognition motif in subunit B was present after the cleavage site of the signal peptide in the protein sequence and was considerably distant from the N terminal region. The mitochondria localization signal prediction score for subunit E was higher than subunit A, although subunit E does not contain a TOM20 recognition site. Proteins with higher numbers of hydrophobic amino acids in their mitochondria signal peptide are known to localize on the mitochondria inner membrane [41]. The signal peptides of subunit A, B, and E all contain a higher proportion of hydrophobic amino acids (Supplementary Figure S3), suggesting the possibility of these subunits localizing at the inner membrane of the mitochondria.

### 2.2. 5-HT3A and 5HT3E Subunits Localize to Mitochondria

**Figure 1:**
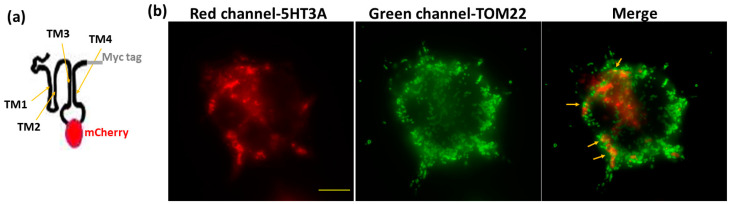
Expression of 5HT3A and 5HT3E subunits in transiently transfected HEK293T cells. (**a**) Schematic of 5HT3A^mCherry-c-Myc^ construct used for transfections. (**b**) Fixed HEK293T cells transiently transfected with 5HT3A^mCherry-c-Myc^ (red channel) and stained with TOM22 antibody (secondary antibody-Alexafluor 488, green channel), and merged image in which yellow arrows point to mitochondria overlapping with subunit 5HT3A^mCherry-c-Myc^ signal. Scale bar is 10 μm.

To see if the 5HT3A and 5HT3E subunits could localize to the mitochondria as predicted, we used fluorescent microscopy to track tagged 5HT3A^mCherry-c-Myc^ and 5HT3E^mCherry-HA^ subunits. The fluorescent tags were inserted between TM3 and TM4 intracellular loop, where they are unlikely to interfere with protein expression or function [23,24], and the c-Myc or HA tag was added at the C terminus (Figure 1a and Figure S4a) [30]. The 5HT3A^mCherry-c-Myc^ and 5HT3E^mCherry-HA^ were transiently transfected into HEK293T cells as these cells do not express endogenous 5-HT_3_ receptors [42]. The images of the HEK293T cells transiently transfected with 5HT3A^mCherry-c-Myc^ show a red fluorescence signal throughout the whole cell except for the nucleus (Figure 1b), similar to previous observations [23,24]. The mitochondria were stained with anti-TOM22 and detected using Alexafluor 488 secondary antibody (green fluorescence) (Figure 1b). The merged images show yellow regions that imply the colocalization of 5HT3A^mCherry-c-Myc^ with mitochondria (Figure 1b). The HEK293T cells transiently transfected with the 5HT3E^mCherry-HA^ subunit generally showed a lower transfection efficiency (Supplementary Figure S4b), reminiscent of the previously seen transfections with 5HT3C^mCherry-FLAG^ [24]. Despite this, the red signal occurred throughout the cells, and there was some overlap in the merged images, indicating potential colocalization with the mitochondria (Supplementary Figure S4b).

**Figure 2:**
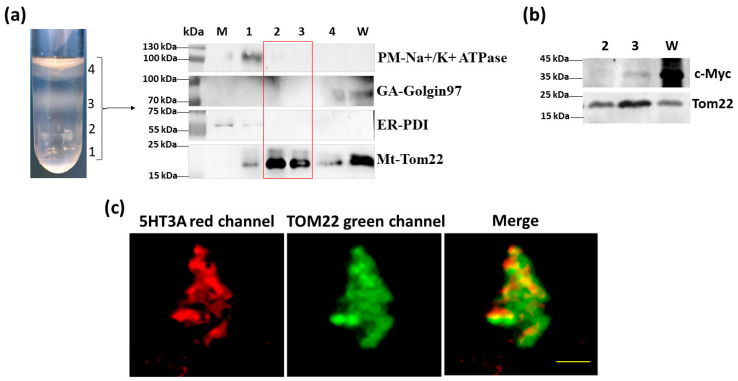
5HT3A subunits are present on mitochondria isolated from transfected HEK293T cells. (**a**) Mitochondria fractions separated in OptiPrep–sucrose gradient (top to bottom order in overlaid crude mitochondria pellet: 10%, 30%, and 50%) and the immunoblots of the whole cell lysate (W), microsome fraction (M) and mitochondrial fractions (1–4) probed with markers for plasma membrane (PM; Na^+^/K^+^ ATPase), endoplasmic reticulum (ER; disulfide isomerase, PDI), Golgi apparatus (GA; Golgin 97), and mitochondria (Mt; TOM22). Full-length immunoblots are shown in Supplementary Figure S10. (**b**) Immunoblot of mitochondrial fractions 2 and 3, isolated from cells transfected with 5HT3A^mCherry-c-Myc^ and probed with c-Myc and TOM22 antibodies. Full-length immunoblots are shown in Supplementary Figure S11a,b. (**c**) Partially dispersed pelleted mitochondrial fraction 3 isolated from 5HT3A^mCherry-c-Myc^-transfected cells. Red channel shows m-Cherry signal, while green channel shows TOM22 antibody detected with Alexafluor 488 with yellow signal overlap in the merged image. Scale bar is 5 μm.

Intracellular mitochondria were isolated via centrifugal fractionation from the 5HT3A^mCherry-c-Myc^-transfected HEK293T cells (Supplementary Figure S5a), and a fraction was purity-assessed via immunoblotting (details of the antibodies used are in Supplementary Table S1). Fractions 2 and 3 were enriched in the mitochondria, as shown by the strong signal with the TOM22 antisera and the lack of contamination with the other tested markers of organelles where 5-HT_3_ receptors are likely to be expressed (Figure 2a). Since 5HT3A^mCherry-c-Myc^ contains the C terminal c-Myc epitope tag, we probed for its presence via immunoblotting. Fraction 3 was enriched in the mitochondria and positive for c-Myc, while fraction 2 contained less signal, indicating that mitochondrial enrichment is associated with the presence of 5HT3A^mCherry-c-Myc^ proteins (Figure 2b). The fluorescence signal of the mitochondrial pellets from fraction 3 that were probed with the anti-TOM22 primary antibody and detected with the Alexafluor 488 antibody (green signal) showed a red fluorescence signal, indicating the presence of 5HT3A^mCherry-c-Myc^ subunits in these pelleted fractions (Figure 2c). We undertook a similar set of experiments with HEK293T cells transfected with 5HT3E^mCherry-HA^ and observed that the isolated mitochondria fraction 3 was enriched in HA and TOM22 signal (Supplementary Figure S4c). Fluorescence microscopy imaging of this fraction revealed the presence of a red signal indicative of 5HT3E subunits (Supplementary Figure S4d). Together, these results indicate that the 5HT3A and 5HT3E subunits can independently occur on mitochondria.

### 2.3. Localization of 5-HT_3_ Receptor Subunits to the Inner Mitochondrial Membrane

**Figure 3:**
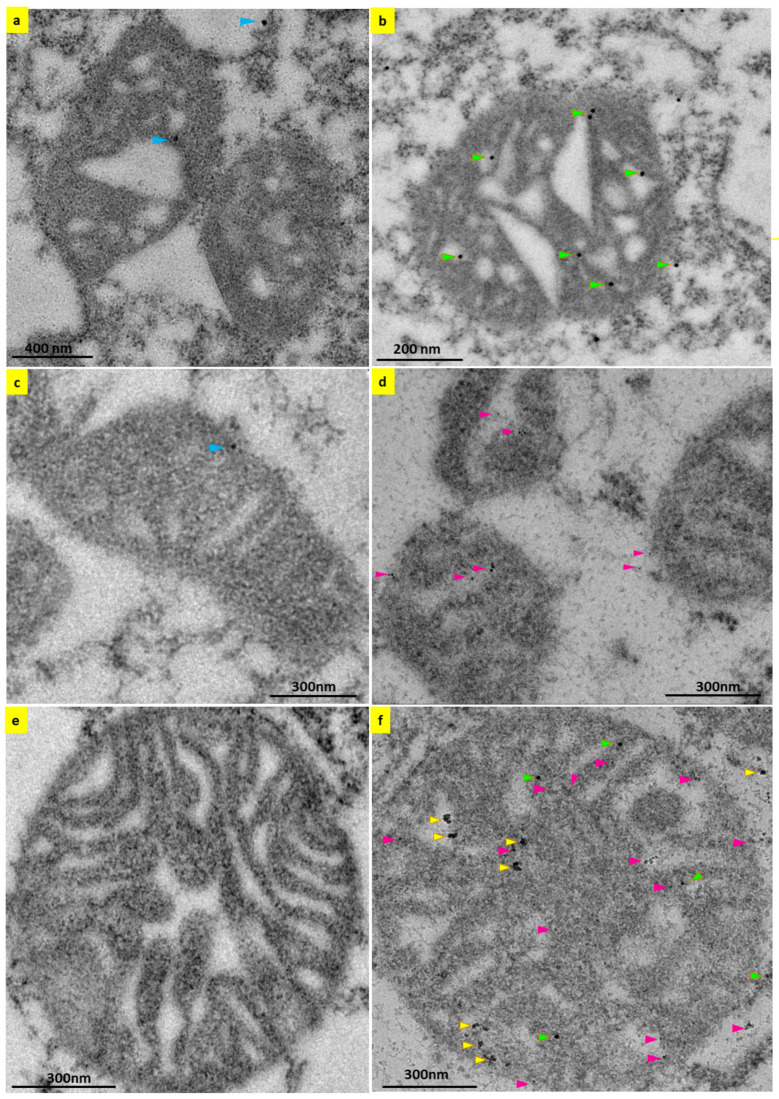
5-HT_3_ receptor subunits present on the inner membrane of mitochondria. Transmission electron micrograph images of mitochondria in 5HT3A^mCherry-c-Myc^ (**a**,**b**) or 5HT3E^mCherry-HA^ (**c**,**d**) and 5HT3A^c-Myc^+ 5HT3E^mCherry-HA^ (**e**,**f**) transiently transfected HEK293T cells. (**a**) Negative control of mitochondria probed with secondary mouse 15 nm gold nanoparticles only, and gold particles are pointed to with blue-colored arrow heads. (**b**) Mitochondria probed with mouse c-Myc antibody to detect 5HT3A^mCherry-c-Myc^ using secondary mouse 15 nm gold nanoparticles. (**c**) Negative control of mitochondria probed with secondary rabbit 6 nm gold nanoparticles. (**d**) Mitochondria probed with rabbit mCherry antibody to detect 5HT3E^mCherry-HA^ using secondary rabbit 6 nm gold nanoparticles. (**e**) Negative control of mitochondria probed with secondary rabbit 6 nm and secondary mouse 15 nm gold nanoparticles. (**f**) Mitochondria probed with primary mouse c-Myc antibody to detect 5HT3A^c-Myc^ and rabbit mCherry antibody to detect 5HT3E^mCherry-HA^ using secondary mouse 15 nm and rabbit 6 nm gold nanoparticles. Gold nanoparticles that stained 5HT3A^mCherry-c-Myc^, 5HT3E^mCherry-HA^, and 5HT3A^c-Myc^ + 5HT3E^mCherry-HA^ subunits are pointed to with green, maroon, and yellow arrowheads, respectively. Scale bars are indicated in each figure.

The mitochondria-targeting peptide sequences of the 5HT3A and 5HT3E subunits were predicted to target these subunits to the mitochondrial inner membrane. To see if this was the case, we used transmission electron microscopy to examine the transfected HEK293T cells. We probed the sections with anti-c-Myc or mCherry antisera and detected these antibodies using secondary antibodies conjugated with gold nanoparticles (Supplementary Table S2). In order to confirm the antibody specificity of the gold nanoparticles, we undertook negative controls, probing with only the secondary antibodies conjugated with 15 nm or 6 nm gold nanoparticles, and a negligible amount (one or two gold particles) of nanoparticles were detected in the background when compared to the positive control (Figure 3a,c). During post-translational activity, the endoplasmic reticulum harbors 5-HT_3_ receptors [35], where the strong fluorescent signal in isolated endoplasmic reticulum microsomal fractions from HEK293T cells transiently transfected with 5HT3A^mCherry-c-Myc^ occurs (Supplementary Figure S6a,b). Therefore, we used the endoplasmic reticulum as a positive control to determine the specificity of the combination of a primary c-Myc antibody and a secondary 15 nm gold antibody in transfected HEK293T cells. Positive staining of the 15 nm gold nanoparticles at distinct sites on the endoplasmic reticulum confirmed that we can detect 5HT3A^mCherry-c-Myc^ subunits in transfected cells (Supplementary Figure S6c). In addition to their presence on the endoplasmic reticulum, 5-HT_3_ receptors were also present on the plasma membrane, where we could also detect 5HT3A^mCherry-c-Myc^ subunits in the HEK293T cells transiently transfected with 5HT3A^mCherry-c-Myc^ (Supplementary Figure S7a).

Furthermore, we detected 5HT3A^mCherry-c-Myc^ subunits on the mitochondria in addition to the plasma membrane in adjacent cells transiently transfected with 5HT3A^mCherry-c-Myc^ (Supplementary Figure S7a). Looking closer, we observed 15 nm gold nanoparticle staining at the inner membranes of the mitochondria, supporting 5HT3A^mCherry-c-Myc^ subunit localization inside the organelle (Figure 3b). Similarly, in cells transfected with 5HT3E^mCherry-HA^, the 5HT3E subunit localizes at the inner membranes of the mitochondria, as shown by 6 nm gold nanoparticle staining (Figure 3d). Typically, the mitochondria in cells transiently transfected with 5HT3A^mCherry-c-Myc^ were stained with three to five gold nanoparticles (15 nm), and those in cells transfected with 5HT3E^mCherry-HA^ were stained with two to three gold nanoparticles (6 nm) per mitochondria. (Supplementary Figure S8). We quantitated the number of gold particles per mitochondria from three mitochondria from four separate cells for each set of transfection experiments and demonstrated that gold particles in the mitochondria of cells transfected with subunit E alone were significantly lower than the gold particles in the cells transfected with subunit A (Supplementary Figure S8). These results support the predictions of the mitochondria-targeting signal and the function of the hydrophobic amino acid residues in the mitochondrial localization of the 5-HT_3_ receptors (Table 1 and Supplementary Figures S1–S3). Further, these findings suggest that subunit A has a role in localizing other subunits to different cellular compartments.

Since subunit A is important for functional 5-HT_3_ receptor formation [2,3,4], we examined whether co-expressing both the A and E subunits in the cells altered their presence on the mitochondria. The co-expression of both subunits (5HT3A^c-Myc^+5HT3E^mCherry-HA^) revealed more than twenty gold nanoparticles per mitochondria (Figure 3f). Moreover, we could identify the presence of just the A (15 nm) or E (6 nm) subunit, or heteromeric complexes in which the gold nanoparticles overlapped on the mitochondria’s inner membrane (Figure 3f). Quantification indicated that the A subunit and colocalized A and E subunits were at similar levels (Supplementary Figure S8). This is the first indication of the presence of possible subunit A and E heteromers at the inner membranes of the mitochondria.

**Figure 4:**
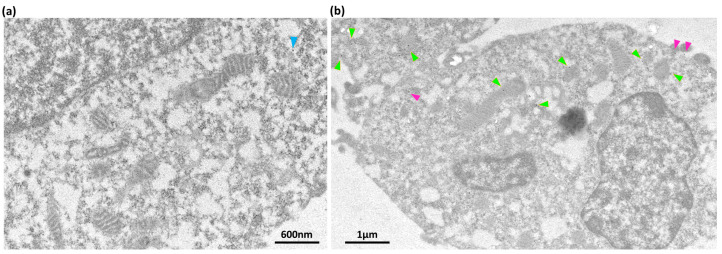
5HT3A subunit on SH SY5Y mitochondria and cell membrane. (**a**) Transmission electron micrograph image of SH SY5Y cells (negative staining) probed with anti-mouse antisera (secondary) tagged with 15 nm gold nanoparticles to detect non-specific binding of the antibody. Scale bar 600 nm. (**b**) Transmission electron micrograph image of SH SY5Y cells probed with mouse 5HT3A antibody (primary) and anti-mouse antisera (secondary) tagged with 15 nm gold nanoparticle to detect native 5HT3A subunits. The 15 nm gold nanoparticles on the mitochondria and plasma membrane are indicated by green and maroon arrowheads, respectively. Scale bar 1 μm. Antibody details can be found in Supplementary Table S2.

To determine whether 5-HT_3_ receptors can occur on the mitochondria of cells that naturally express 5-HT_3_ receptors, we choose the SH SY5Y cell line, a human neuronal cell that expresses endogenous 5-HT_3_ receptors [43]. Using transmission electron microscopy and 5HT3A antisera, 5-HT_3_ receptors were detected on the inner membrane region of SH SY5Y cells (Figure 4). Therefore, it appears that the localization of the 5-HT_3_ receptors occurs due to the mitochondrial signal peptide and not because of their overexpression in HEK293T cells.

### 2.4. Cell-Free Mitochondria Contain 5HT3A Subunits

**Figure 5:**
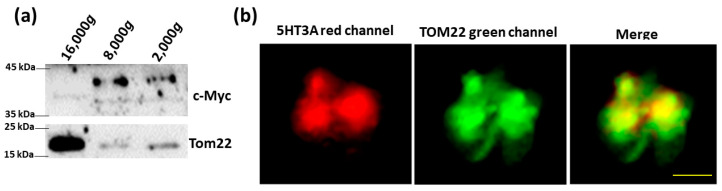
Cell-free mitochondria carry 5HT3A subunits. (**a**) Immunoblots of cell-free mitochondria collected from cell culture media of HEK293T cells transiently transfected with 5HT3A^mCherry-c-Myc^ and probed with c-Myc epitope tag antibody (c-Myc) to detect 5HT3A subunits and TOM22 antibody to detect mitochondrial membranes. Full-length immunoblots can be viewed in Supplementary Figure S11c. (**b**) Pelleted clumps of cell-free mitochondria collected from the cell culture media of 5HT3A^mCherry-c-Myc^-transfected HEK293T cells. Red channel shows 5HT3A, and green channel shows TOM22 antibody-stained mitochondria. The merged image shows mitochondria with 5HT3A subunits. Scale bar is 5 μm.

Interestingly, mitochondria can be found outside the cell, and these cell-free mitochondria are involved in several diseases [44]. Since 5-HT_3_ receptors are clinical targets, we investigated whether the mitochondria that escape cells carry 5-HT_3_ receptors. Therefore, we collected cell-free mitochondria from the cell culture media of HEK293T cells transfected with 5HT3A^mCherry-c-Myc^ (Supplementary Figure S5b). The cell-free mitochondria were positive for TOM22 and the c-Myc-epitope tag in immunoblots (Figure 5a). Further, these pelleted samples also showed red fluorescence co-locating with green fluorescence from the TOM22 detected by Alexafluor 488 (Figure 5b), implying that cell-free mitochondria do carry 5HT3A subunits. In addition, using transmission electron microscopy, we detected 5HT3A subunits on mitochondria spotted in the extracellular areas of cells transiently transfected with 5HT3A (Supplementary Figure S7b). These mitochondria are possibly cell-free mitochondria.

However, cell-free mitochondria isolated from the cell culture media of 5HT3E^mCherry-HA^ transfected HEK293T cells did not show any HA label on immunoblots or a red fluorescence signal (Supplementary Figure S4c,d). Since the transfection efficiency of the subunits’ 5HT3E^mCherry-HA^ is lower than 5HT3A^mcherry-myc^, such as 5HT3C^mcherry-FLAG^ [24], we checked whether the isolated intracellular mitochondria fractions from these cells contained 5HT3E subunits. Purified mitochondria fractions showed the presence of the HA-epitope tag protein, and these fractions also showed a red fluorescence signal, supporting the localization of subunit E with the mitochondria (Supplementary Figure S4c,d). However, the HA-epitope tag protein and red fluorescence signal were not observed in the cell-free mitochondria (Supplementary Figure S4d).

### 2.5. 5-HT_3_ Receptors Modulate Mitochondrial Membrane Potential

**Figure 6:**
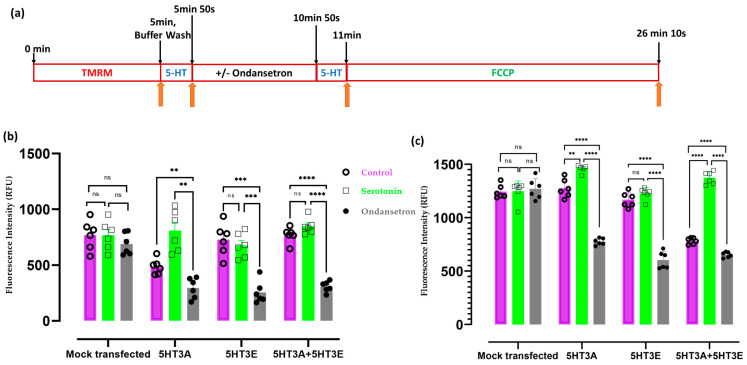
5-HT_3_ receptors affect mitochondrial membrane potential. (**a**) Schematic timeline representation of the TMRM and drug treatment for both HEK293T cells and isolated mitochondria. (**b**) Mitochondrial membrane potential in HEK293T cells (250 cells/well) mock-transfected or transiently transfected with 5HT3A or 5HT3E or both 5HT3A+5HT3E subunits and treated with a vehicle (Milli-Q water; control), serotonin alone, or ondansetron plus serotonin. The fluorescence values obtained when measuring TMRM fluorescence were corrected for the cell number. (**c**) Mitochondrial membrane potential in purified mitochondria (500 µg/well) obtained from HEK293T cells mock-transfected or transfected with 5HT3A or 5HT3E or both 5HT3A+5HT3E subunits. Mitochondria were treated with a vehicle (Milli-Q water; control) or serotonin alone or ondansetron plus serotonin. For both b and c, all samples were treated with FCCP after drug treatment as a depolarization control, and data were analyzed via two-way ANOVA, followed by Dunnett’s multiple comparisons test (*n* = 6, ** *p* < 0.01, *** *p* < 0.001, **** *p* < 0.0001, ns—not significant).

Since 5-HT_3_ receptor subunits can locate to the mitochondria, we examined how the different subunits influenced the mitochondrial membrane potential in transiently transfected HEK293T cells in response to serotonin in the presence or absence of the 5-HT_3_ receptor antagonist ondansetron (Figure 6). Serotonin was applied at a concentration of 30 μM to intact cells or preparations of mitochondria as this concentration was previously shown to elicit good responses in mammalian cell studies transfected with either homomeric or heteromeric 5-HT_3_ receptor combinations [30]. Cells transfected with the 5HT3A^c-Myc^ subunit showed a small increase in the fluorescence signal in response to 30 μM serotonin. This is possibly due to the lower value of the control treatment in the 5HT3A transfection, as it is not seen in either the 5HT3E or the 5HT3A + 5HT3E heteromeric combination or the mock-transfected control cells (Figure 6b). Pre-treatment with 3 nM of ondansetron significantly suppressed the fluorescence signal in response to serotonin in cells transfected with 5HT3A, 5HT3E, or the combination of 5HT3A + 5HT3E; however, no change was observed in the mock-transfected cells (Figure 6b).

Although a response from the cells to 5-HT was observed, it was not clear whether the 5-HT_3_ receptor subunits on the mitochondria are responsive to the drugs. Therefore, we isolated mitochondria from HEK293T cells transiently transfected with 5HT3A, 5HT3E, or the combination of 5HT3A+5HT3E subunits or the mock-transfected cells (as a control) and undertook a similar set of experiments. The isolated mitochondria were functional as shown by their functional efficacy via glutamate-malate- and succinate-driven respiration for both basal (state 2) and ADP (state 3)-induced states (Supplementary Figure S9). The mitochondria isolated from the cells transfected with 5-HT_3_ receptor subunits showed a reduced response to serotonin following pre-treatment with ondansetron that was not seen in the mitochondria from mock-transfected cells (Figure 6c). Mitochondria from cells transfected with the 5HT3A subunit showed a slight increase in the fluorescence signal in response to 30 μM serotonin, while no response was seen in the mitochondria from cells transfected with the 5HT3E subunit (Figure 6c). Although there was a significant increase in the response to 30 μM serotonin in the mitochondria isolated from cells transfected with the 5HT3A+5HT3E subunits (Figure 6c), this appears to have been due to the puzzling lower control response that occurred in these mitochondria. Taken together, these results suggest that ondansetron blocks the mitochondrial membrane potential of mitochondria with the 5HT3A or 5HT3E subunit and the 5HT3A + 5HT3E subunits.

### 2.6. 5-HT_3_ Receptor Alters the Oxygen Consumption Rate of Mitochondria

**Figure 7:**
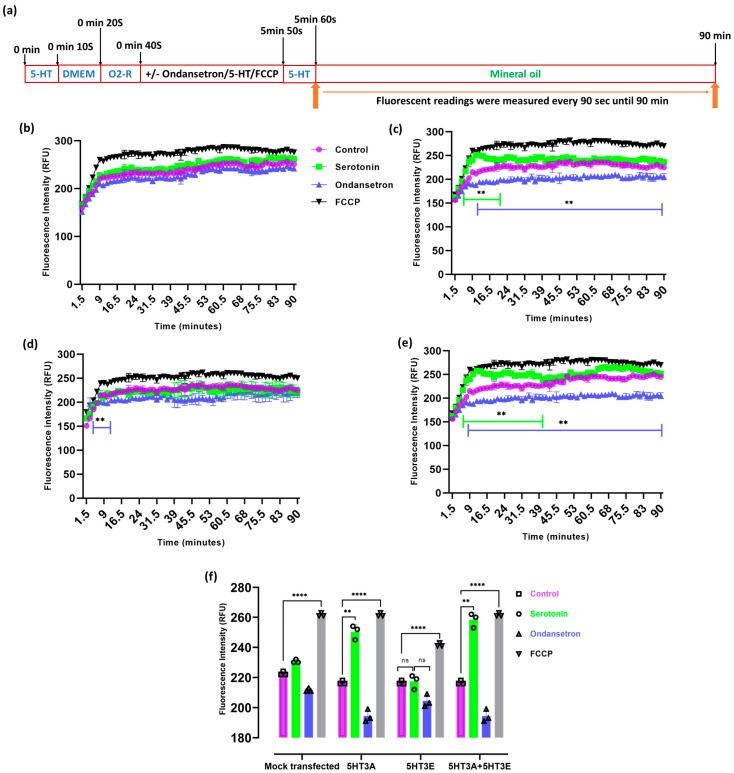
5-HT_3_ receptor subunits affect cellular oxygen consumption rates. (**a**) Schematic of the drug treatment of HEK293T cells and fluorescence measurement timings. (**b**–**e**) Oxygen consumption rate in HEK293T cells (250 cells per well) mock-transfected or transiently transfected with 5HT3A, 5HT3E, or both 5HT3A and 5HT3E subunits. HEK293T cells were treated with vehicle (Milli-Q water; control) or serotonin (30 μM) alone, ondansetron (3 nM) and serotonin (30 μM), or FCCP alone (10 μM). (**b**) HEK293T cells mock transfected. (**c**) HEK293T cells transiently transfected with 5HT3A. (**d**) HEK293T cells transiently transfected with 5HT3E. (**e**) HEK293T cells transiently transfected with 5HT3A + 5HT3E. (**f**) Comparison of oxygen consumption rates of mock-transfected and 5HT3A and/or 5HT3E transfected HEK293T cells at 12 min timepoint. All assays were analyzed by two-way ANOVA, followed with Dunnett’s multiple comparison test (*n* = 3; ** *p* < 0.01, **** *p* < 0.0001, ns—not significant, green lines indicate time course differences between control and serotonin and blue lines between control and ondansetron in (**b**) to (**e**)).

As we detected 5-HT_3_ receptors on the inner mitochondrial membrane (Figure 3) and 5-HT_3_ specific agonists have been shown to raise relative oxygen consumption rates [36], we investigated the oxygen consumption of HEK293T cells transiently transfected with 5HT3A and/or 5HT3E subunits (Figure 7a).

Oxygen consumption steadily increased in the mock-transfected HEK293T cells during the monitoring period, whereas the cells transfected with the 5HT3A and 5HT3A+5HT3E subunits exhibited a significant increase in their oxygen consumption rate under serotonin (30 µM) exposure (Figure 7b–e). Uncoupling the mitochondria with FCCP induced high rates of oxygen consumption in all cells. A significant difference (*p* < 0.01) in oxygen consumption was observed between the vehicle-treated (Milli-Q water) samples and samples treated with 30 µM serotonin in subunit-5HT3A- or subunit-5HT3A + 5HT3E-transfected cells (Figure 7c,e), while the subunit-E-transfected cells showed no significant difference between the vehicle-treated samples and samples treated with 30 µM serotonin (Figure 7d,f). In subunit-5HT3A- or 5HT3A+5HT3E-transfected cells, oxygen consumption was reduced upon pre-treatment with ondansetron (*p* < 0.01) (Figure 7c,e). The serotonin-induced increases in the oxygen consumption rates were the most similar between the 5HT3A- and 5HT3A+5HT3E-transfected cells, while the 5HT3E-transfected cells had a minimal response to serotonin, implying that the A subunit is required. A minimum change in the oxygen consumption rate occurred in mitochondria containing subunit E alone (Figure 7b–e). A further comparison of oxygen consumption among the different groups revealed significant differences in oxygen consumption between the vehicle (control) and serotonin with or without ondansetron treatments at the 12 min time point. A significant increase in the oxygen consumption rate was observed at the 12 min time point in the 5HT3A- or 5HT3A+5HT3E-transfected cells under serotonin, which was not observed in the 5HT3E- or mock-transfected cells (Figure 7f). Ondansetron pretreatment significantly suppressed the serotonin-induced oxygen consumption in the 5HT3A- and 5HT3A+5HT3E-expressing cells upon serotonin exposure during the monitoring period. However, subunit-E-expressing HEK293T cells showed a no effect under serotonin exposure at the 12 min time point.

**Figure 8:**
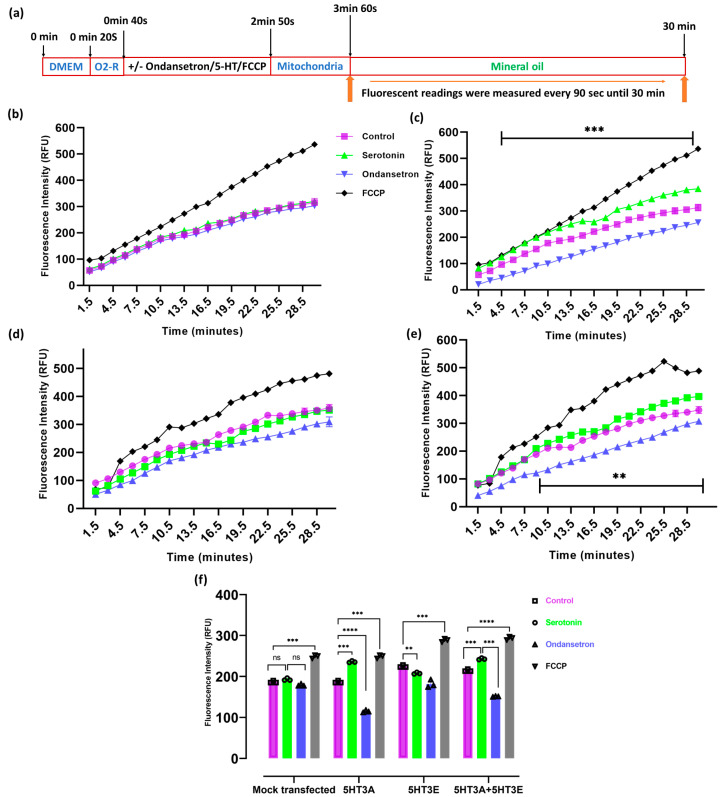
5-HT_3_ receptor subunits affect mitochondrial respiration. (**a**) Schematic of the drug treatment of isolated mitochondria and fluorescence measurement timings. (**b**–**e**) Oxygen consumption rate in purified mitochondria (500 μg per well) isolated from HEK293T cells mock-transfected or transiently transfected with 5HT3A or 5HT3E or both 5HT3A and 5HT3E subunits. Mitochondria were treated with vehicle (Milli-Q water; control) or serotonin (30 μM) alone or ondansetron (3 nM) plus serotonin (30 μM) or FCCP alone (10 μM). (**b**) Mitochondria from mock-transfected HEK293T cells. (**c**) Mitochondria from HEK293T cells transiently transfected with 5HT3A. (**d**) Mitochondria from HEK293T cells transiently transfected with 5HT3E. (**e**) Mitochondria from HEK293T cells transiently transfected with 5HT3A + 5HT3E. (**f**) Comparison of oxygen consumption rates of isolated mitochondria at 12 min timepoint. All assays were analyzed via two-way ANOVA, followed with Dunnett’s multiple comparison test (*n* = 3; ** *p* < 0.01, *** *p* < 0.001, **** *p* < 0.0001, ns—not significant, black lines in parts c and e highlight the time course significant differences between control and serotonin or ondansetron treatments).

To determine if these effects on oxygen consumption mediated by 5-HT_3_ receptors were directly related to their presence on the mitochondria, we measured the oxygen consumption in isolated mitochondria. The uncoupler FCCP induced high rates of oxygen consumption in all mitochondrial preparations (Figure 8). Mitochondria isolated from mock-transfected or transiently transfected HEK293T cells were exposed to serotonin in the presence or absence of ondansetron, and their extracellular oxygen consumption rates were analyzed. Oxygen consumption steadily increased in the mock-transfected mitochondria during the monitoring period (Figure 8b). Notably, the mitochondria with 5HT3A and 5HT3A+5HT3E subunits exhibited a significant increase in oxygen consumption under serotonin (30 µM) exposure, while those containing only 5HT3E subunits did not (Figure 8c–e). Similar to the HEK293T cells, the mitochondria with 5HT3A and 5HT3E showed reduced oxygen consumption in response to serotonin if pre-treated with ondansetron (Figure 8). Further, the comparison of the oxygen consumption among the different groups at the 12 min time point highlights the significant increase in oxygen consumption in the 5HT3A- or 5HT3A+5HT3E-subunit-containing mitochondria that is abrogated by pre-treatment with ondansetron (Figure 8f).

## 3. Discussion

It is well established that 5-HT_3_ receptors, like other members of the ligand-gated ion channel family, are found on the plasma membrane, where binding and electrophysiology studies have shown them to be functional [21,23,45,46]. The receptor subunits are processed to the plasma membrane from the endoplasmic reticulum where the subunits assemble into receptor complexes [28,47] and following desensitization, the receptors are internalized to be recycled or degraded [48,49,50,51,52,53]. Therefore, the presence of 5-HT_3_ receptor subunits was expected to be on the endoplasmic reticulum, vesicles, plasma membrane, and endosomes. Surprisingly, 5-HT_3_ receptors were reported on mitochondria where they maintain the homeostasis of mitochondrial [Ca^2+^] and reactive oxygen species (ROS) and the ATP generation efficiency in mouse cardiomyocytes under hypoxic conditions [36]. This finding intrigued us, so we sought to see whether human 5-HT_3_ receptor subunits can occur in the mitochondria.

Many mitochondrial proteins are imported into mitochondria by translocator complexes in the mitochondria membranes. Targeting peptides are present at the N-terminal region of the proteins, which helps in mitochondrial localization [54]. These protein signals are classified into an N-terminal-cleavable targeting signal (pre-sequence) and a non-cleavable internal targeting signal [55]. Firstly, via in silico analysis, we identified near the N-terminal 4- and 43-amino-acid-long mitochondria-targeting peptides for subunit A and E protein sequences, respectively. We also confirmed that the A and E subunits have a more hydrophobic sequence in this region, potentially favoring their import into the mitochondria [1,56].

Using 5-HT_3_ receptor subunits incorporating fluorescent and/or epitope tags, we explored their intracellular localization. We fractionated the endoplasmic reticulum and mitochondria and identified that the tagged 5HT3A and 5HT3E subunits are associated with these fractions. To investigate their precise cellular location, we made use of the tags to detect the receptors in the immunogold nanoparticle labeling of transiently transfected HEK293T cells using transmission electron microscopy. Here, we could clearly see 5HT3A subunits associated with the endoplasmic reticulum and the plasma membrane as expected. We also ascertained their association with the mitochondria and their localization at mitochondrial inner membranes (Figure 3). The dual immunogold labeling of mitochondria with both 5HT3A and 5HT3E subunits revealed a lower number (~3) of gold nanoparticles on the mitochondria with subunit E alone; however, a higher number (~6 to 20) of gold nanoparticles was observed on mitochondria when both the 5HT3A+5HT3E subunits were present. This suggests the existence of possible subunit A and E heteromers, and possibly indicates that subunit A assists in localizing higher numbers of 5HT3E subunits onto the mitochondrial inner membrane.

SH SY5Y cells endogenously express 5-HT_3_ receptors, and these appear to be involved in H_2_O_2_-induced oxidative stress [43]. A prior study indicated the involvement of the 5-HT_3_ receptor in regulating the homeostasis of mitochondria in mouse cardiomyocytes [36]. Here, we show for the first time that SH SY5Y contain 5-HT_3_ receptors on the inner membranes of their mitochondria. Together, these results support the possible involvement of mitochondria-localized 5-HT_3_ receptors in an oxygen-dependent metabolism.

These observations raise the possibility that 5-HT_3_ receptor ligands can affect intracellular mitochondria. We show that the natural agonist serotonin induces increases in oxygen consumption and alters membrane potential in cells expressing 5-HT_3_ receptors and that these events are negated if the 5-HT_3_ receptor antagonist ondansetron is present. Further, this effect is at least in part a direct effect of the receptors being present on the mitochondria, as similar results are seen with isolated mitochondria. These findings in 5HT3A- and 5HT3E-harboring mitochondria not only confirm the 5-HT_3_ receptor subunit location in the mitochondria but underscore their regulatory effect in mitochondrial function. However, a limitation to the study is that these experiments were only performed using transfected HEK293T cells and one 5-HT_3_-receptor-specific ligand. It would be of interest to test whether the mitochondria from cells such as SH SY5Y or others also show a direct response to 5-HT_3_ receptor ligands.

To form a functional 5-HT_3_ receptor, subunit A is essential due to the ligand-binding region in its receptor that forms at the interface of two A subunits to create the orthosteric binding site [3,4,29,31,57,58,59,60,61]. Subunit E can be expressed individually in the cells and can participate in the heteromer activity [30,32]; however, it is unclear if it can form functional homomers [30,62]. In our study, we saw an effect of ondansetron on HEK293T cells transfected with 5HT3E alone and on mitochondria isolated from these cells in both membrane potential and oxygen consumption assays. These preparations did not respond to serotonin treatment alone, but as ondansetron reduced the serotonin response, it does suggest the possibility of an ondansetron binding site on subunit E homomers. This may be due to ondansetron blocking any serotonin entry at the 5HT3E orthosteric binding site or the possible allosteric activity of ondansetron at subunit E homomers [3,31,60,61].

Several studies have discussed the glucose-5-HT_3_ receptor association in the gut–brain axis [46,63,64,65,66]. The expression levels of 5-HT_3_ receptors on vagal afferent neurons are altered by varying concentrations of extracellular D-glucose in the rat gastrointestinal tract [67,68,69]. The localization of the 5-HT_3_ receptor at different organs has implications for signal pathways. For example, 5-HT_3_ receptors in the colon induced colonic inflammation through substance P (SP) and the neurokinin-1 (NK1) receptor, and 5-HT_3_ receptors signaling the neural crest (NC) alter the development of the visceral organs, including the lower urinary tract (LUT) [70,71]. At the cellular level, most studies show functional receptors at the plasma membrane, although one study indicated the presence of a functional 5-HT_3_ receptor on mitochondria; however, the exact location was not pinpointed [36]. Mitochondria are associated with processes of generating reactive oxygen species (ROS), inflammation, cell death, aging, and neuronal regeneration [72,73]. The finding of the present study implies that the changes in the oxygen consumption rate in 5-HT_3_ receptor-containing mitochondria might influence the generation of reactive oxygen species. Mitochondria also play important roles in lipid and carbohydrate metabolism [74,75]. Possibly, the 5-HT_3_ receptors present in mitochondria may participate in this type of response via changes in the mitochondria associated ATP production or carbohydrate metabolism.

The presence of functionally active 5-HT_3_ receptors in the mitochondria also raises the possibility that they are involved in mitochondria-mediated lipid metabolism and cell death. Previous studies indicated that serotonin and 5-HT_3_-receptor-specific drugs (e.g., ondansetron) regulate the lipid concentrations and cell death activity in cells [76,77,78]. For example, blocking 5-HT_3_ receptors with the antagonist ondansetron induces an increase in cholesterol and triacylglycerol concentrations in mouse models [78]. In another study, increasing serotonin levels induced cell death in human colon carcinoma cells, while ondansetron abolished cell death [76].

Furthermore, in this study, we report the existence of cell-free mitochondria harboring 5-HT_3_ receptors. The existence of cell-free mitochondria and cell-free mitochondrial DNA in the circulatory system is becoming topical [79,80]. There are suggestions that cell-free mitochondrial DNA may be the reason behind depressive disorders [81] and contribute to the chronic inflammation seen in type 2 diabetes [82]. On the other hand, cell-free mitochondria have been considered as potential therapeutic interventions for central nervous system (CNS) disorders [81,83]. In addition, artificial mitochondria transfer prevented apoptosis in human T lymphocytes [84,85]. Earlier studies refer to “Mitochondrial donation” and “Mitochondria replacement therapies”, which have been used to treat homoplasmic conditions and mitochondrial optic neuropathies such as Leber’s hereditary optic neuropathy disorders [86,87,88,89,90]. Our results raise the possibility that some cell-free mitochondria may contain 5-HT_3_ receptor subunits and flow in the circulatory system, where they may respond to 5-HT and 5-HT_3_ receptor antagonists.

## 4. Conclusions

The presence of 5HT3A and 5HT3E subunits and the possible formation of 5-HT_3_AE receptor heteromers on mitochondria in HEK293T cells raises the possibility that mitochondria are the actual targets of 5-HT_3_ receptor ligands at times. Transmission electron microscopy revealed the subunits on the inner mitochondrial membrane in transiently transfected HEK293T cells and an 5HT3A subunit on the inner mitochondrial membranes of SH SY5Y cells which naturally express 5-HT_3_ receptors. 5-HT_3_ receptors are ligand-gated ion channels, and their position on the inner membrane suggest that they directly affect mitochondrial metabolic functions. Membrane potential and oxygen consumption rate analyses of mitochondria carrying 5-HT_3_ receptors revealed that they respond to serotonin and that the response is suppressed when the antagonist ondansetron is present. These results pave a path for future research to explore the direct relation between 5-HT_3_ receptors and mitochondrial signaling.

## 5. Materials and Methods

### 5.1. Chemicals and Media

Molecular-biology-grade reagents were purchased: Poly-D-Lysine (Millipore, Burlington, MA, USA), protease inhibitor cocktail (Roche, Basel, Switzerland), Dulbecco’s Modified Eagle’s Medium (DMEM)-L-glutamine (Gibco DMEM-F12), fetal bovine serum (FBS: Australian source: Corning, Corning, NY, USA), Roswell Park Memorial Institute—1640 (RPMI) (Gibco), Ampicillin (Sigma-Aldrich, St. Louis, MO, USA), Gentamycin (Gold Bio-G), paraformaldehyde (Sigma), Luria broth (MP biomedicals, Tokyo, Japan), pure-yield plasmid miniprep system protocol (Promega, Madison, WI, USA), Gateway LR Clonase II enzyme mix (Invitrogen, Waltham, MA, USA), OmniMAX 2 T1 chemically competent cells (Invitrogen), SOC Media (Invitrogen) OptiMEM (Gibco), Trypan blue (Sigma), Lipofectamine 3000 transfection reagent (Thermo Fisher, Waltham, MA, USA), methanol (AjaxFinechem, New South Wales, Australia), ethanol (AjaxFinechem), Triton X-100 (Sigma), OptiPrep density gradient medium (Sigma-Aldrich), tetramethylrhodamine methyl ester (TMRM) (Merk, Rahway, NJ, USA), carbonyl cyanide 3-chlorophenylhydrazone (FCCP) (Sigma), mannitol (Mallinckrodt, St. Louis, MO, USA), KH_2_PO_4_ (Astral Scientific, Taren Point, Australia), MgCl_2_ (Sigma), ethylenediaminetetraacetic acid (EDTA) (Astral scientific), 4-(2-hydroxyethyl)-1-piperazineethanesulfonic acid (HEPES) (Bio Basic), uranyl acetate (EMS microscopy academy, Hatfield, PA, USA), lead citrate (EMS microscopy academy), ethanol, EM-grade (Emsure Supelco), LR White resin (EMS), serotonin hydrochloride (Sigma), serotonin (Sigma Aldrich), ondansetron hydrochloride (Sigma), and oxygen-sensing fluorophore (Abcam, Cambridge, UK).

### 5.2. In Silico Analyses of Localization Signals Present in 5-HT_3_ Receptor Subunits

5HT3A (AAP35868.1), 5HT3B (NP_006019.1), 5HT3C (NP_570126.2), 5HT3D (AAI01092.1), and 5HT3E (NP_872395.2) subunit sequences were submitted to standalone web-interface programs to predict if they contained localization signals. The software used were: TPpred 2.0 (https://tppred2.biocomp.unibo.it/welcome/default/index) [39], MitoFates (https://mitf.cbrc.pj.aist.go.jp/MitoFates/cgi-bin/top.cgi) [40], SignalP (https://services.healthtech.dtu.dk/service.php?SignalP-5.0) [38], and Eukaryotic Linear Motif (http://elm.eu.org/) [37]. All the software listed above were last accessed on 2 February 2022. To analyze the hydrophobic amino acid residues in the signal peptide of the 5-HT_3_ receptor subunit protein sequence, NetWheels software [91] was used and last accessed on 16 January 2023.

### 5.3. 5-HT_3_ Receptor Subunit Constructs

We used the 5HT3A constructs containing mCherry fluorescent protein between the TM3 and TM4 domains (G395-G396 residues) as previously described [24]. We also prepared a 5HT3E construct containing mCherry via the Gibson cloning procedure as previously described, using the HA-epitope-tagged 5HT3E construct [30] as the template. The fluorescent protein was inserted so that it would be expressed between the G396 and V397 residues in the intracellular loop between TM3 and TM4 of the E subunit. The primers used for confirming the sequence identity of the plasmids are listed in Supplementary Table S3, and the identities and correct sequences were confirmed via sequencing (Micromon Monash University). Entry constructs with correct sequences were cloned into destination vector pcDNA-DEST40 using a Gateway LR Clonase II enzyme mix (Thermo Fisher). Constructs with only epitope tags were named 5HT3A^c-Myc^ and 5HT3E ^HA^, and constructs with both fluorescent tags and epitope tags were named 5HT3A^mCherry-c-Myc^ and 5HT3E^mCherry-HA^. The destination vectors were transformed into DH5α chemically competent *Escherichia coli* cells, grown on ampicillin (100 µg/mL) selection LB agar plates, and positive colonies were confirmed via colony PCR (Supplementary Table S3). Positive bacterial colonies were picked and regrown in overnight LB culture in the presence of ampicillin, and plasmid DNA was isolated using Promega plasmid miniprep kit, following the manufacturer’s protocol. Bulk plasmid DNA for transfections was isolated and purified using MACHEREY-NAGEL maxiprep protocol [92].

### 5.4. Cell Culture and Transfections

HEK293T (ATCC CRL-3216)-adherent cells were grown in DMEM-GlutaMAX culture media containing L-glutamine supplemented with 5% fetal bovine serum (FBS). The cells were grown in a humidified atmosphere of 95% O_2_ and 5% CO_2_ in a CO_2_ incubator at 37 °C. The cells were tested with PCR Mycoplasma detection kit (TOKU-E MO34-02) and mycoplasma-free cells, usually between passage number 8 to passage number 20, were used for the experiments. The HEK293T cells were seeded at a density of 1 × 10^4^ cells in 6-well plates containing poly-D-Lysine-coated cover slips (22 × 22 mm, Menzel Glaser # 11728691). After 24 h, the cells were transfected with plasmid constructs of the 5HT3A and/or 5HT3E subunits using Lipofectamine 3000 transfection reagent, according to the manufacturer’s protocol.

For fluorescent and electron microscopy imaging purposes, we transfected HEK293T cells with 5HT3A^mCherry-c-Myc^ or 5HT3E^mCherry-HA^ individually for homopentameric receptors; for heteromeric receptor observations, we transfected the HEK293T cells with both 5HT3A^c-Myc^ and 5HT3E^mCherry-HA^ at 1:9 ratio. This 1:9 ratio was previously determined to yield a more equitable distribution of the fluorescent signal [24]. For the mitochondrial membrane potential assays and oxygen consumption assays, we transfected the HEK293T cells with 5HT3A^c-Myc^ or 5HT3E^HA^ constructs individually, and for heteromeric receptor observations, we transfected the HEK293T cells with both 5HT3A^c-Myc^ and 5HT3E^HA^ at a 1:9 ratio.

### 5.5. Isolation of Mitochondrial Fractions

The HEK293T cells (7.5 × 10^8^ cells/T75 flask) were harvested in phosphate-buffered saline (PBS) (137 mM NaCl, 2.7 mM KCl, 10 mM Na_2_HPO_4_, 1.8 mM KH_2_PO_4_) and pelleted in 50 mL tubes 48 h after transfection. The fractional isolation procedure was based on the gradient centrifugation protocol developed by Shapovalov et al. [93], as depicted in Supplementary Figure S4a. The cells were homogenized in ice-cold buffer B1 (250 mM sucrose, 1 mM EDTA, 20 mM HEPES-NaOH pH 7.4) using a 13 × 118 mm (3 mL volume) glass Potter homogenizer (Pyrex #7725T-3) with five repetitions. All centrifugation steps were carried out at 4 °C, using the JA-30.50 Ti rotor in a Beckman Coulter-Avanti JXN-30 centrifuge. Briefly, a low-speed centrifugation at 1000× *g* for 15 min at 4 °C was performed twice to pellet down the nuclear fraction and cellular debris. The supernatant was collected for the further separation of the mitochondria or microsome fractions. Mitochondria were pelleted in a centrifugation step at 17,000× *g* for 30 min. To purify the mitochondria from other cellular contaminants (plasma membrane and endoplasmic reticulum), the pellet was washed, resuspended with ice-cold buffer B2 (1 mM EDTA; 20 mM HEPES-NaOH pH 7.4), loaded onto a 10%, 30%, and 50% three-layer OptiPrep–sucrose gradient, and processed at 100,000× *g* for 1 h, where pure mitochondria banded between the 10% and 30% gradient layers and all fractions were collected separately. The supernatant collected from the 17,000× *g* spin was subjected to 100,000× *g* for 1h to pellet the microsome fraction (membranes), and the supernatant was collected to represent the whole cell soluble protein fraction.

The purity of mitochondrial and other fractions isolated from the HEK293T cells was assessed via immunoblotting, using specific organelle protein markers. The protein concentrations in the fractions were estimated using the Bradford assay [94] with bovine serum albumin (BSA) as a standard, and the absorbance at 595 nm was measured with a CLARIOstar (BMG LABTECH) plate reader. Then, 15 μg protein samples were separated in a 12% SDS-PAGE gel and run at 100 V for 2 h in a running buffer (25 mM Tris, 190 mM glycine, 0.1% SDS) at room temperature. The separated proteins were transferred to Amersham Hybond ECL nitrocellulose membrane (RPN303D) at 60 V for 2.5 h in a transfer buffer (25 mM Tris, 190 mM glycine, 0.1% SDS, 20% methanol) at 4 °C. Blots were blocked in 1× Tris-buffered saline (TBS; 137 mM NaCl, 2.7 mM KCl, 19 mM Tris) containing 3% skimmed milk (blocking buffer) for 1 h, followed by three washes with 1× TBS for 5 min each. After the washing, a primary antibody (Supplementary Table S1) diluted in the blocking buffer at a 1:1000 ratio indicated in Supplementary Table S1, was added to the blot, and the blot was incubated overnight at 2 °C to 5 °C. The blot was then subjected to three washes with 1× TBS for 5 min each, followed by incubation with the secondary antibody for 1 h at room temperature. Finally, the blot was washed four times for 5 min each with 1× TBST (0.1% Tween 20 in TBS). An Amersham ECL Select (RPN2235) Western blotting detection reagent was used to develop the immunoblots in the gel dock system (SYNGENE–G-Box Chemi-XL1, Gene Sys version 1.2.8.0) with a 1.4 Mega Pixel Synoptics camera. Endoplasmic reticulum fractions were detected with antisera to the disulphide isomerase (PDI), and mitochondrial fractions were detected with antisera to the translocase of the outer mitochondrial membrane22 (TOM22). Any plasma membrane or Golgi apparatus contamination of fractions were detected by using antibodies directed against Na^+^/K^+^ ATPase or Golgin 97, respectively. Details about primary and secondary antibodies used in the experiments are provided in Supplementary Table S1.

### 5.6. Isolation of Cell-Free Mitochondria

Cell-free mitochondria were collected from the HEK293T cell culture media (from three T75 flasks). We followed the protocol that isolated the free-floating mitochondria from the cell culture [79], as depicted in Supplementary Figure S4b. HEK293T cells (7.5 × 10^8^ cells/T75 flask) were transfected with 5HT3A and 5HT3E subunit constructs. After 36 h post transfection, the cells were transferred into T75 flasks and incubated for a further 24 h before collecting the cell culture media from the flask without disturbing the cells. This media was spun at 1000× *g* × 10 min to remove dead cells and nucleus debris from the media. This step was repeated twice to make sure that cells or other large organelles were totally removed from the media. We collected the supernatant and spun it at 8000× *g* × 15 min in the first step and collected the pellet containing the intact mitochondria. The supernatant was transferred to a new centrifuge tube and spun at 16,000× *g* × 15 min in the second step, and we collected the pellet that contained the smaller mitochondria. In the third step, the supernatant was transferred to a new centrifuge tube and spun at 40,000× *g* × 1 h, and we collected the pellet containing smaller organelles. The pellets were resuspended in an assay buffer (70 mM sucrose, 220 mM mannitol, 5 mM KH_2_PO_4_, 5 mM MgCl_2_, 1 mM EDTA, 2 mM HEPES) for further processing.

### 5.7. Imaging of the Cells and Purified Mitochondria

#### 5.7.1. Sample Preparation for Fluorescence Imaging

HEK293T cells were seeded onto poly-D-Lysine coated coverslips as described above. Pure mitochondrial fractions (100 μL) isolated from HEK293T cells were spread onto a poly-D-Lysine-coated coverslip and incubated for 3 h at 4 °C to absorb to the glass [95]. The transiently transfected HEK293T cells or mitochondrial fractions on the cover slips were fixed with 4% (*w/v*) paraformaldehyde in PBS. The cells were permeabilized with 0.5% Triton X-100 in PBS. The coverslips were then washed three times for 5 min each with PBS and then blocked overnight in 2% (*w/v*) BSA in PBS at 4 °C [96]. The cells and mitochondrial fractions were then incubated with a primary antibody against TOM22 at a dilution of 1:100 in 2% BSA for 2 h at 4 °C. The coverslips were then washed three times (5 min each) with PBS and incubated at room temperature for 1 h with the Alexafluor 488-anti-mouse conjugated secondary antibody at a dilution of 1:1000. Details of the primary and secondary antibodies used in the experiments are provided in Supplementary Table S1. The coverslips were then washed two times with PBS and mounted with 1× PBS on a glass slide and sealed together with nail polish.

#### 5.7.2. Transmission Electron Microscopy Sample Preparation

HEK293T cells and the pure mitochondria isolated from the transiently transfected HEK293T cells were collected as a pellet (<1 mm sized pellet) and fixed in a freshly prepared primary fixative of 2% paraformaldehyde and 2.5% glutaraldehyde in 1× Sorensen’s phosphate buffer (S-PB) (133 mM Na_2_HPO_4_, 0.133 mM KH_2_PO_4_) and incubated at 4 °C overnight. The next day, the pellet was washed for 10 min in 1× S-PB three times at 4 °C. The S-PB was then removed through a graded ethanol series (30, 50, 70, and 90%) for 10 min each to dehydrate the specimens. The pellet was then transferred into 100% ethanol and incubated for 10 min, and this final dehydration step was performed twice. Next, the pellet was transferred into a series of resin ethanol mixtures, 1:3, 1:1, and 3:1 100% ethanol and 100% London Resin (LR) White resin and incubated for 10 min. The pellet was then transferred into 100% LR White resin, where it was incubated for 10 min, and this step was repeated three times before incubating the pellet overnight at 60 °C. These blocks were trimmed using a diamond knife (Ultra-Microtome, EM UC7 Leica) at 80–100 nm, and sections were placed on 200-mesh thin-bar hexagonal copper grids with a formvar coating (ProScitech, Kirwan, Australia).

For immunogold staining grids, sections were blocked with 0.05 M glycine for 20 min, followed by incubation with 1% BSA in 0.1 M phosphate-buffered saline (PBS) for 30 min. Then grids were then incubated with primary antibody for 1 h. The grids were washed with PBS for 10 min three times before undergoing three 10 min washes with 1% BSA. The grids were then incubated with secondary antibody for 1 h, followed by three 10 min Milli-Q water washes. Both the primary and secondary antibodies were diluted in 1% BSA in PBS. The nonspecific-binding negative staining of sections was performed in parallel with secondary antibody only. Details of the antibodies used in the immunogold staining are provided in Supplementary Table S2. Following antibody staining, the grids were incubated with 2% uranyl acetate at room temperature for 15 min before they were washed in Milli-Q water three times and allowed to dry at room temperature. The grids were then incubated over lead citrate (NaOH pellets were used to reduce CO_2_ in a covered dish to prevent lead precipitation on the grids) for 30 s and finally rinsed in 0.02 M NaOH in recently boiled and cooled Milli-Q water. They were then rinsed again in plain Milli-Q water (10–20 s) two more times. The grids were imaged with a Jeol JEM-2100 transmission electron microscope using an 80 KV electron beam with an exposure time of 2000 ms/frame with the Gatan Orius 200 and AMT Nanosprint 15 cameras.

### 5.8. Mitochondrial Membrane Potential Assays

HEK293T cells (200 cells/well) or mitochondria (500 µg/well) were plated in black-wall, transparent, flat-bottom 96 well plates (CLS3603-48EA, Corning-3603) for membrane potential assays. The HEK293T cells were transfected with either 5HT3A or 5HT3E or the combination of 5HT3A and 5HT3E (5HT3A + 5HT3E) at a 1:9 ratio of construct DNA, using lipofectamine 3000 (Invitrogen) reagent as described in Section 5.4. The mitochondrial membrane potential was measured using an assay incorporating the tetramethyl rhodamine methyl ester (TMRM) fluorescent dye adapted from BMG Labtech protocol (number AN175). Briefly, cells or mitochondria were loaded with TMRM at 150 nM in the assay buffer (80 mM NaCl, 75 mM KCl, 25 mM D-glucose, 25 mM HEPES, pH 7.4) at 37 °C for 5 min, the cells were washed four times in PBS, and signal detected was carried out on a BMG CLARIOstar microplate reader (excitation: 544 nm and emission: 590 nm, bottom reading with 50 flashes per well). An assay buffer containing 30 μM serotonin was applied to the cells for 10 s, and fluorescence readings were taken followed by washing with the assay buffer. The 5-HT_3_ receptor antagonist ondansetron (0.3 nM in Milli-Q water) replaced the assay buffer and was incubated for a period of 5 min, followed by the application of 30 μM of serotonin for 10 s. Responses to 30 μM of serotonin were recorded at the end of the 5 min ondansetron treatment. After these readings, 10 μM of FCCP was added to all the wells, incubated for 15 min, and washed four times in PBS. Following the uncoupling of the mitochondria, the fluorescent signal was measured. All data are expressed as the total TMRM fluorescence minus the TMRM fluorescence after FCCP treatment. A schematic of the drug treatment timeline is depicted in Figure 5a.

### 5.9. Oxygen Consumption Rate (OCR) Measurements

HEK293T cells (200 cells/well) or mitochondria (500 µg/well) were plated in black-wall, transparent, flat-bottom 96-well plates, as described above. The culture medium was changed from serum-containing DMEM to serum-free DMEM during the OCR measurement. The oxygen-sensing fluorophore at 10 µL per 150 µL of sample was added to measure the extracellular O_2_ consumption. All the samples were first treated with a vehicle (Milli-Q water) and 30 μM OF serotonin for 10 s. the samples were THEN washed with PBS three times, and 150 µL of fresh, serum-free DMEM media was loaded onto the cells. Then, 10 µL of O_2_ consumption reagent was loaded on to the samples, followed by 0.3 nM of ondansetron and 10 μM of FCCP onto the respective sample wells. After incubation for 4 min 50 s, 30 μM of serotonin was loaded onto the designated samples. After adding treatment compounds to the samples, 100 µL of prewarmed mineral oil at 37 °C was loaded on top of the reagent mix in 96 wells (blanks were made with a reagent and media but no cells), and the plate was promptly sealed. The responses to 30 μM of serotonin and 0.3 nM ondansetron and 10 μM FCCP treatment were recorded after, as depicted in the schematic timeline (Figure 7a). The extracellular O_2_ consumption signal was measured at 1.5 min intervals for 90 min on a CLARIOSTAR microplate reader at 37 °C (Excitation: 380 nm and Emission: 650 nm, bottom reading with LP-TR Dichroic). To measure extracellular O_2_ consumption in the mitochondria, we added 100 µL of extracellular O_2_ consumption reagent reconstituted in measurement buffer (250 mM sucrose, 15 mM KCl, 30 mM KH_2_PO_4_, 5 mM MgCl_2_, 1 mM EDTA, pH 7.4) at 1:10 ratio to each sample well. Then, 30 μM of serotonin and 10 μM of FCCP were added to the respective sample wells. Then, 50 µL (500 µg) of diluted, isolated mitochondria were added to each test well. For ondansetron treatment readings, the mitochondria incubated for 5 min with 0.3 nM of ondansetron loaded in the wells, followed by 30 μM serotonin treatment. For state 2 and state 3 respiration analyses, 50 µL of substrate (25 mM and 12.5/12.5 mM for succinate and glutamate/malate final concentration, respectively) was added without or with ADP (1.65 mM final concentration) in a measurement buffer. Then, 100 µL mineral oil, prewarmed at 30 °C, was loaded on top of the reagent mix (blanks were made with the reagent and media but no mitochondria and substrate), and the was plate promptly sealed. The extracellular O_2_ consumption signal was measured at 1.5 min intervals for 30 min, as described above. A schematic of the drug and substrate treatment timeline is depicted in Figure 8a. The values of the treated sample fluorescent readings were corrected with respect to the blanks (baseline slopes in the absence of cells).

### 5.10. Data Analysis

Fluorescence microscopy and electron microscopy image data were analyzed with ImageJ software [97]. Data from the mitochondrial membrane potential and oxygen consumption assays were represented as mean ± SEM and analyzed by a two-way ANOVA test followed by Dunnett’s multiple comparison test. *p* values < 0.05 were regarded as significant. Antibody labeling data were statistically analyzed using a two-way ANOVA, followed by Šidák multiple comparison tests. All analyses conducted made using GraphPad Prism 9.5.0 software.

## Figures and Tables

**Table 1:** 5-HT_3_ receptor subunit localization signal peptide predictions.

Software	Subunit	Cleavage Site	Signal Peptide	Prediction Score	Reference
TPpred2 (3.0)	5HT3A	-	-	0.996	[39]
	5HT3B	-	-	1.000	
	5HT3C	-	-	0.954	
	5HT3D	-	-	0.973	
	5HT3E	43 amino acid	1–43 amino acid	0.771	
MitoFates	5HT3A	32 amino acid	2–6 amino acid	0.018	[40]
	5HT3B	49 amino acid	-	0.000	
	5HT3C	25 amino acid	-	0.006	
	5HT3D	39 amino acid	-	0.000	
	5HT3E	71 amino acid	-	0.023	
SignalP-5.0	5HT3A	27 amino acid	1–21 amino acid	0.916	[38]
	5HT3B	21 amino acid	1–21 amino acid	0.978	
	5HT3C	27 amino acid	1–25 amino acid	0.993	
	5HT3D	24 amino acid	1–23 amino acid	0.992	
	5HT3E	27 amino acid	1–25 amino acid	0.983	
ELM	5HT3A	26 amino acid	1–24 amino acid	-	[37]
	5HT3B	27 amino acid	1–26 amino acid	-	
	5HT3C	32 amino acid	1–29 amino acid	-	
	5HT3D	24 amino acid	1–23 amino acid	-	
	5HT3E	30 amino acid	1–28 amino acid	-	

## Data Availability

All data are reported in the manuscript and in the Supplementary Materials.

## Supplementary Materials

The following supporting information can be downloaded at: https://www.mdpi.com/article/10.3390/ijms24098301/s1. References [39,40,79,91,93,98,99,100,101,102,103,104,105] are cited in the Supplementary Materials.
