# Supplementary material for: 5-HT3 Receptors on Mitochondria Influence Mitochondrial Function
Source: Int J Mol Sci. 2023 May 5;24(9):8301. doi: 10.3390/ijms24098301 (PMC10179570; doi:10.3390/ijms24098301)
Supplement: Supplementary file 1 [file ijms-24-08301-s001.zip › ijms-2317745-supplementary.pdf]

Supplementary information for

5-HT3 receptors on mitochondria influence mitochondrial function

Santosh T. R. B. Rao[1,2], Ilona Turek[1,2], Julian Ratcliffe[1,3], Simone Beckham[4], Cassandra Cianciarulo[1,2], Siti S B M Y Adil[2], Christine Kettle[1,2], Donna R Whelan[1,2], Helen R. Irving[1,2]*.

1 La Trobe Institute for Molecular Science, La Trobe University, PO Box 199, Bendigo VIC 3552, Australia. s.tata@latrobe.edu.au, i.turek@latrobe.edu.au, c.cianciarulo@latrobe.edu.au, c.flens@latrobe.edu.au, d.whelan@latrobe.edu.au, h.irving@latrobe.edu.au.

2 Department of Rural Clinical Sciences, La Trobe University, PO Box 199, Bendigo VIC 3552, Australia.

3 Bio-Imaging platform, La Trobe University, Kingsbury Dr, Bundoora VIC 3086, Australia. j.ratcliffe@latrobe.edu.au.

4 Regional Science Operations, La Trobe University, PO Box 199, Bendigo VIC 3552, Australia.

* Correspondence: h.irving@latrobe.edu.au; Tel.: +61354447551

Contents of this document

Supplementary Table S1. Antibodies used to detect cell organelles in the purified fractions.

Supplementary Table S2. Antibodies used in electron microscopy imaging.

Supplementary Table S3. Primers used for Gateway cloning and sequence verification.

Supplementary Figure S1. Mitochondria localization signal prediction for 5-HT3 receptor subunits using TPPred2 software.

Supplementary Figure S2. Tom20 recognition motif prediction in signal peptide sequence of 5-HT3 receptor subunits using MitoFates software.

Supplementary Figure S3. Hydrophobic amino acids identification in the N-terminal region of the 5-HT3 receptor subunits protein sequences using NetWheels software.

Supplementary Figure 4. Schematic diagram of intracellular and cell-free mitochondria isolation from transiently transfected HEK293T cells.

Supplementary Figure S5. 5HT3E subunit location on intracellular and extracellular mitochondria.

Supplementary Figure S6. 5HT3A$^{mCherry-myc}$ subunits on the endoplasmic reticulum.

Supplementary Figure S7. 5HT3A$^{mCherry-myc}$ subunits on the plasma membrane and mitochondria.

Supplementary Figure S8. Profiles for glutamate malate (12.5mM) (a) and succinate (25mM) (b) driven respiration of mitochondria isolated from HEK293T cells.

Supplementary Figure S9. Comparison of the particle density of immunogold particles on mitochondria.

Supplementary Figure S10. Full-length immunoblots images corresponding to Figure 2a and Supplementary Figure 6a.

Supplementary Figure S11. Full-length immunoblots images corresponding to immunoblot sections are shown in Figure 2b, Figure 4a, and Supplementary Figure 5a.

Supplementary Table S1. Antibodies used to detect cell organelles or 5-HT3A/E subunits in the purified fractions.

**Cell organelle/ 5HT3 subunit**	**Antibody (Molecular weight of a target protein)**	**Dilution**	**Host specie**	**Catalogue number, Company**	**References**
Plasma membrane	Na^+/K^+ ATPase (113kDa)	1:1000	Rabbit	Ab76020, Abcam	[1]
Golgi apparatus	Golgin 97 (97kDa)	1:1000	Mouse	A-21270, Invitrogen	[2]
Endoplasmic Reticulum	Disulphide isomerase (PDI) (57kDa)	1:1000	Rabbit	P7496, Sigma Aldrich	[3]
Mitochondria	Translocase of outer mitochondrial membrane22 (TOM22) (22kDa)	1:1000 (immunoblotting)/1:100 (imaging)	Mouse	T6319, Sigma Aldrich	[4]
Secondary antibody	Alexafluor Plus 488 IgG	1:1000	Goat (anti-mouse)	A32723, Invitrogen	[5]
Secondary antibody	Anti-rabbit IgG	1:1000	Goat (anti-rabbit)	A-6154, Sigma Aldrich	[6]
Secondary antibody	Anti-mouse IgG	1:1000	Goat (anti-mouse)	A-0168, Sigma Aldrich	[7]
Secondary antibody	Anti-mouse IgG	1:1000	Rabbit (anti-mouse)	A-9044, Sigma Aldrich	[8]
5-HT3A	c-Myc tag	1:1000	Mouse	2276, Cell Signaling Technology	[9]
5-HT3E	HA tag	1:500	Rabbit	A190-138A, ThermoFisher	

Supplementary Table S2. Antibodies used in electron microscopy imaging.

Subunit	Antibody (Molecular weight of a target protein)	Dilution	Host species	Catalogue number, Company	Reference
5HT3A	c-Myc tag	1:70	Mouse	2276, Cell Signaling Technology	[9]
	5HT3A	1:70	Mouse	MA5-31770	
	Mouse 15 nm gold nano particles	1:20	Mouse	JA115-022, Aurion	
5HT3E	mCherry	1:70	Rabbit	PA5-34974, Invitrogen	[10]
	Rabbit 6 nm gold nano particles	1:20	Rabbit	JA106-011, Aurion	

Supplementary Table S3: Primers used for Gateway cloning and sequence verification.

Name of the primer	Primer sequence (5′-3′)	Primer purpose
3E For FP	ACCCACCTGCCCGGTATGGTGAGCAAGGGCGAG	Fluorescent protein overhang inserts into HTR3E
3E Rev FP	CTCTGGCTCCTTCACGTACAGCTCGTCCATGCC	Fluorescent protein overhang inserts into HTR3E
3E For	CTGTACGTGAAGGAGCCAGAGGTATCAGCAGG	HTR3E linearization
3E Rev	CACCATACCGGGCAGGTGGGTGGG	HTR3E linearization
h5-HT3A short Forward	CTCCTGGGCTACTCGGTCT	Sequencing
h5-HT3A short Reverse	GCACAATGAAGATGGTCTCG	Sequencing
h5-HT3Ea Forward	ATCCTTCAGACCCATGGAGA	Sequencing
h5-HT3Ea Reverse	ACTGGGCACGAGAAGGTTTA	Sequencing
T7 promoter Forward	TAATACGACTCACTATAGGG	Sequencing
Fluorescent Protein Reverse	GCGCGGGTCTTGTAGTTG	Sequencing
mCherry Reverse	CCCATGGTCTTCTTCTCCAT	Sequencing

Rev: Reverse; For: Forward; FP: Fluorescent protein.

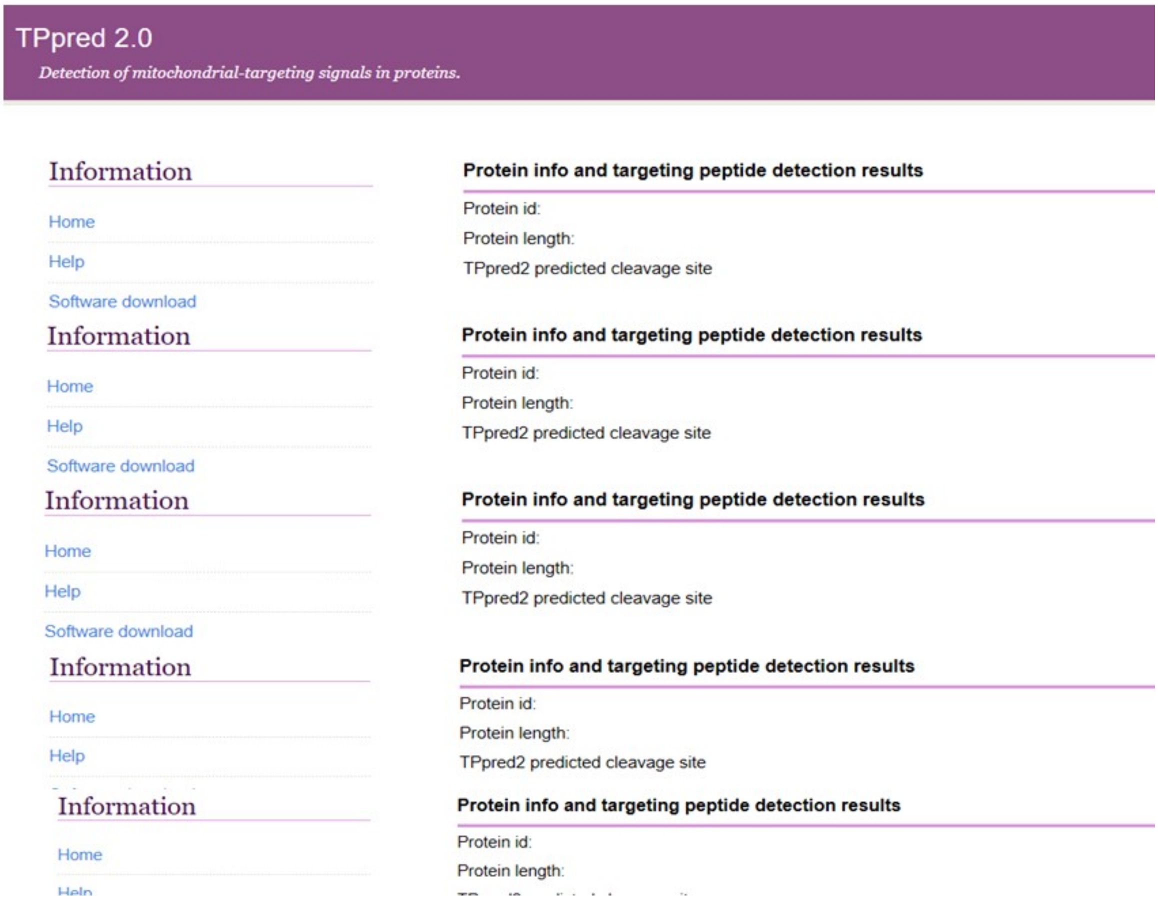

Supplementary Figure S1. Mitochondria localization signal prediction for 5-HT$_3$ receptor subunits using TPpred2 software. **(a)** 5HT3A (AAP35868.1), **(b)** 5HT3B (NP_006019.1), **(c)** 5HT3C (NP_570126.2), **(d)** 5HT3D (AAI01092.1), and **(e)** 5HT3E (NP_872395.2) subunit protein sequences were analysed with TPpred2 software [11]. Cleavage sites and prediction scores are shown, and the entire protein sequence of subunit E is shown with mitochondria targeting peptide region amino acid residues highlighted in orange and mature protein amino acid residues highlighted in blue.

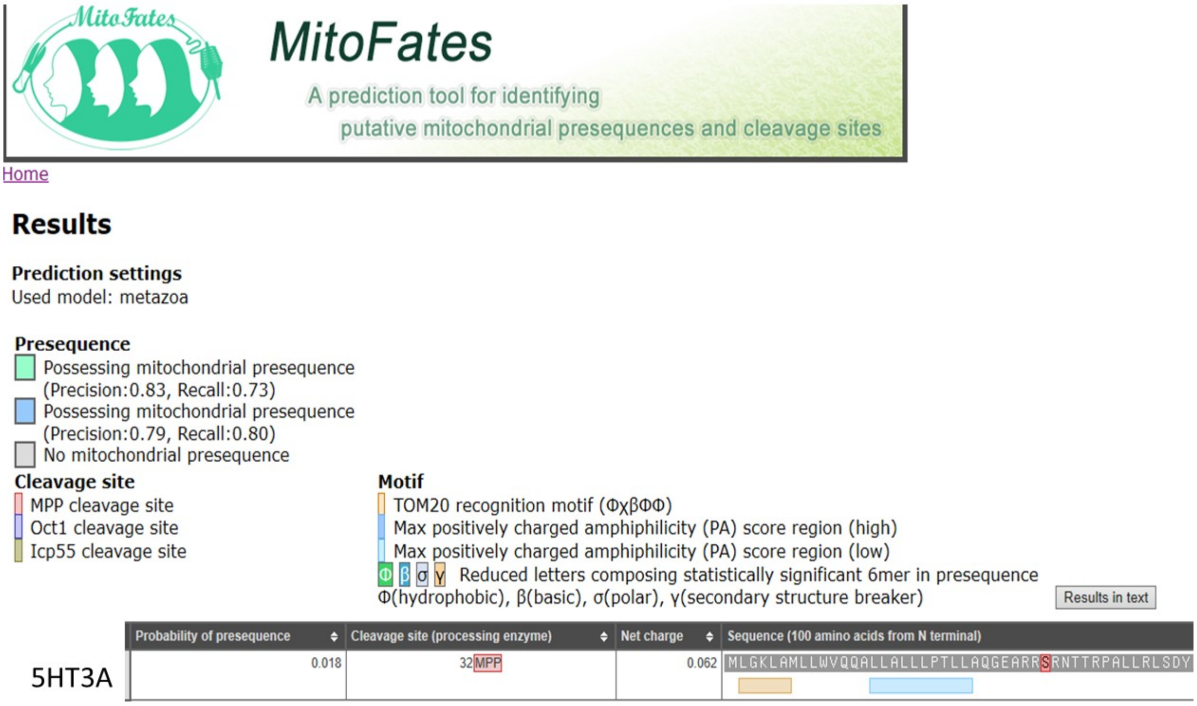

Supplementary Figure S2. Tom20 recognition motif prediction in signal peptide sequence of 5-HT$_3$ receptor subunits using MitoFates software. (a) 5HT3A (AAP35868.1), **(b)** 5HT3B (NP_006019.1), **(c)** 5HT3C (NP_570126.2), **(d)** 5HT3D (AAI01092.1), and **(e)** 5HT3E (NP_872395.2) subunit protein sequences were analysed with MitoFates software [12] for the presence of mitochondrial pre-sequence TOM20 recognition motif. Scores for probability of pre-sequence, position of the cleavage site and net charge are represented in the first 3 columns. TOM20 recognition motif in the subunit protein sequence is denoted by an orange box under the predicted motif residues. The blue box denotes a highly positive and amphiphilic amino acid residues region in the protein sequence. The amino acid residue at the cleavage site is highlighted in red

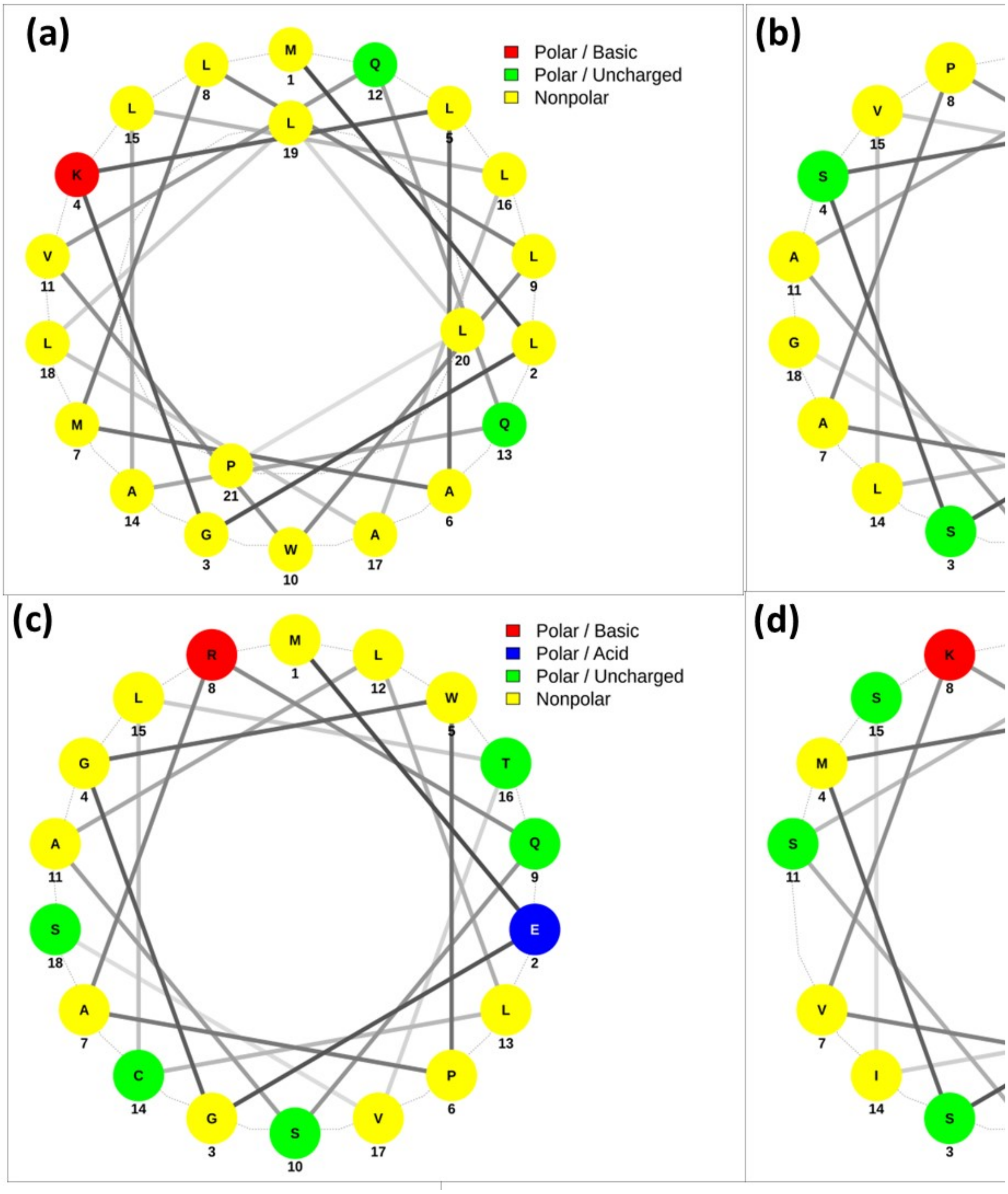

Supplementary Figure S3. Hydrophobic amino acids identification in the N-terminal region of the 5-HT$_3$ receptor subunit protein sequences using NetWheels software. (a) 5HT3A (AAP35868.1), **(b)** 5HT3B (NP_006019.1), **(c)** 5HT3C (NP_570126.2), **(d)** 5HT3D (AAI01092.1), and **(e)** 5HT3E (NP_872395.2) subunit protein sequences were analysed using NetWheels software [13] to identify hydrophobic amino acid residues in their signal peptide region. Amino acids in the figure are listed in coloured circles according to their chemical nature. Polar basic amino acids are in red circles, polar acidic in blue circles, polar uncharged in green circles and nonpolar or hydrophobic amino acids are in yellow circles.

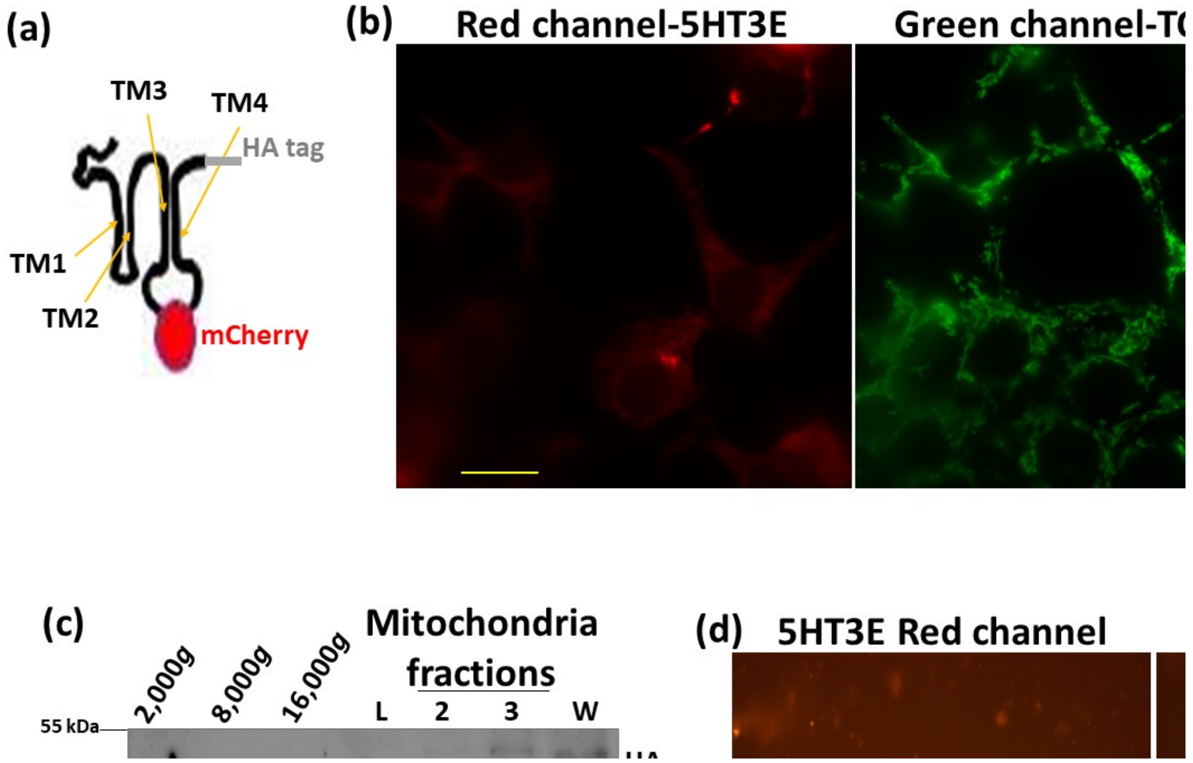

Supplementary Figure S4. 5HT3E subunit location on intracellular and extracellular mitochondria. **(a)** Schematic of 5HT3E$^{mCherry\text{-}HA}$ construct used for transfections. **(b)** Fixed HEK293T cells transiently transfected with 5HT3E$^{mCherry\text{-}HA}$ (red channel) and stained with TOM22 antibody (secondary antibody - Alexafluor 488, green channel), merged image where yellow arrows point to mitochondria overlapping with 5HT3E$^{mCherry\text{-}HA}$ signal. **(c)** Immunoblots of mitochondria purified via OptiPrep-sucrose gradient method or cell-free mitochondria isolated from cell culture media were probed with anti-HA epitope tag antibody, and anti-Translocase of outer mitochondrial membrane22 (TOM22) antibody. In the blot, numbers 2 and 3 represent mitochondrial fractions, W represents whole cell fraction, 2000g, 8000g and 16000g refer to cell free mitochondria samples collected at these centrifugation rates, L denotes ladder in the figure. Full length immunoblots are shown in Supplementary Figure 11d and e. **(d)** Microscopic images of purified intracellular mitochondria or cell-free mitochondria with 5HT3E$^{mCherry\text{-}HA}$ detected in the red channel. Scale bars for images are 10μm. Antibody details can be found in Supplementary Table 1.

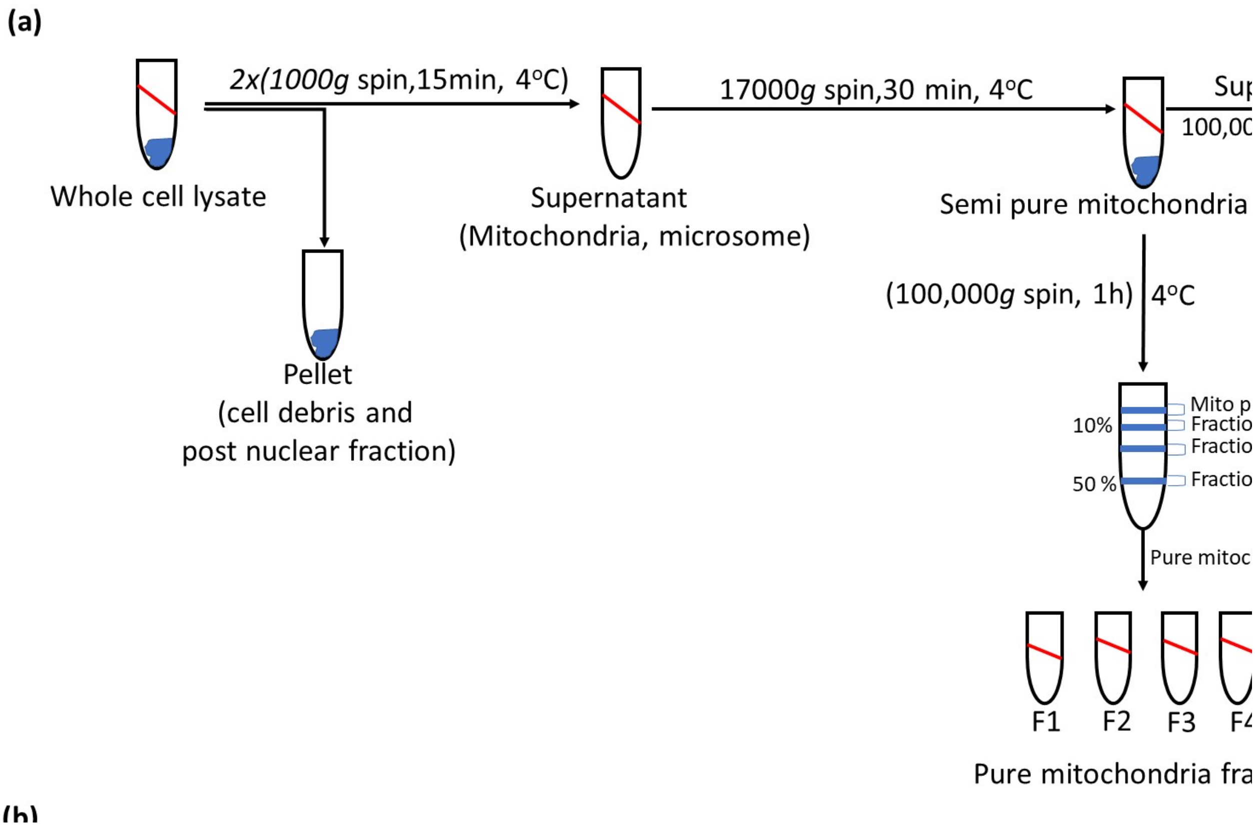

Supplementary Figure S5. Schematic diagram of isolation of intracellular and cell-free mitochondria from transiently transfected HEK293T cells. (a) OptiPrep-sucrose gradient-based mitochondria purification protocol adapted from Shapovalov *et al*. [2]. A detailed description is in the methods section 5.5. **(b)** Cell-free mitochondria isolation from HEK293T cell culture media protocol adapted from Al Amir Dache *et al* [14]. A detailed description is in the methods section 5.6.

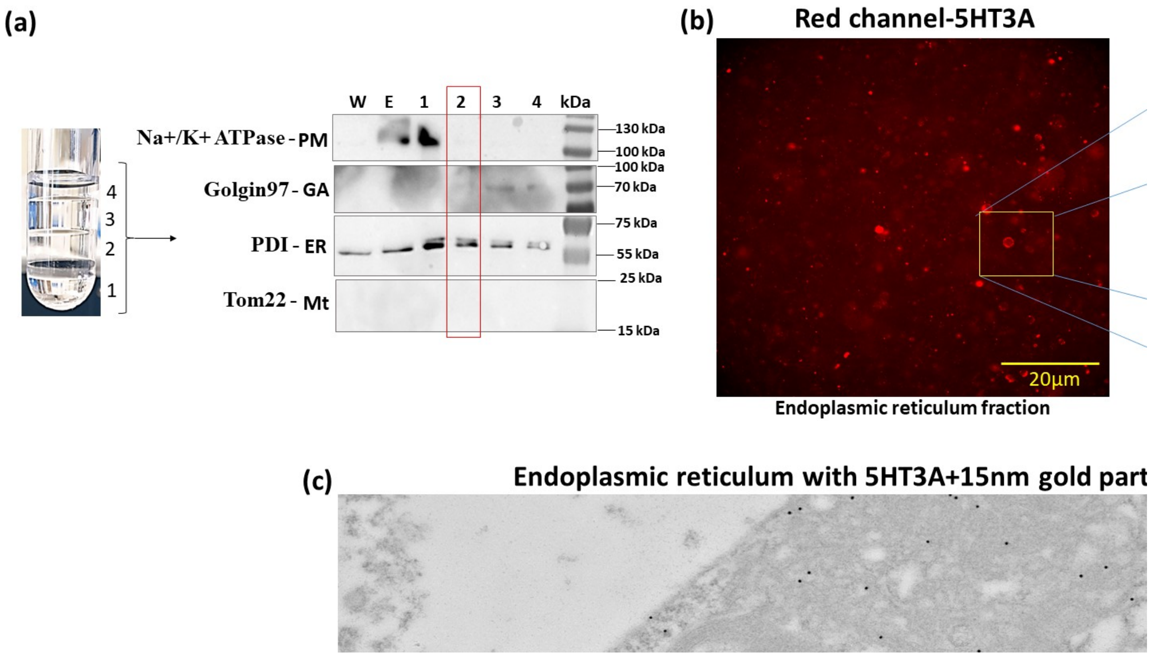

Supplementary Figure S6. 5HT3A$^{mCherry\text{-}c\text{-}Myc}$ subunits on the endoplasmic reticulum. (a) Immunoblots of separate endoplasmic reticulum fractions (1 (10%), 2 (30%), 3 (50%), and 4 (overlaid microsome fraction)) were probed with markers for plasma membrane (PM; Na^+/K^+ ATPase), endoplasmic reticulum (ER; disulphide isomerase; PDI), Golgi apparatus (GA; Golgin 97), and mitochondria (Mt; TOM22). Full length immunoblots are shown in Supplementary Figure 10. **(b)** Micrograph of purified endoplasmic reticulum fraction 2 from 5HT3A$^{mCherry\text{-}c\text{-}Myc}$ transiently transfected HEK293T cells showing red channel fluorescence from 5HT3A$^{mCherry\text{-}c\text{-}Myc}$ subunits on the surface of the endoplasmic reticulum microsome. Scalebar 20µm and for the enlarged image 5µm. **(c)** Transmission electron micrograph images of endoplasmic reticulum probed with mouse c-Myc antibody (primary) and 15 nm gold nanoparticle tagged anti-mouse antisera (secondary) to detect 5HT3A$^{mCherry\text{-}c\text{-}Myc}$. Scalebar 600nm.

(a)

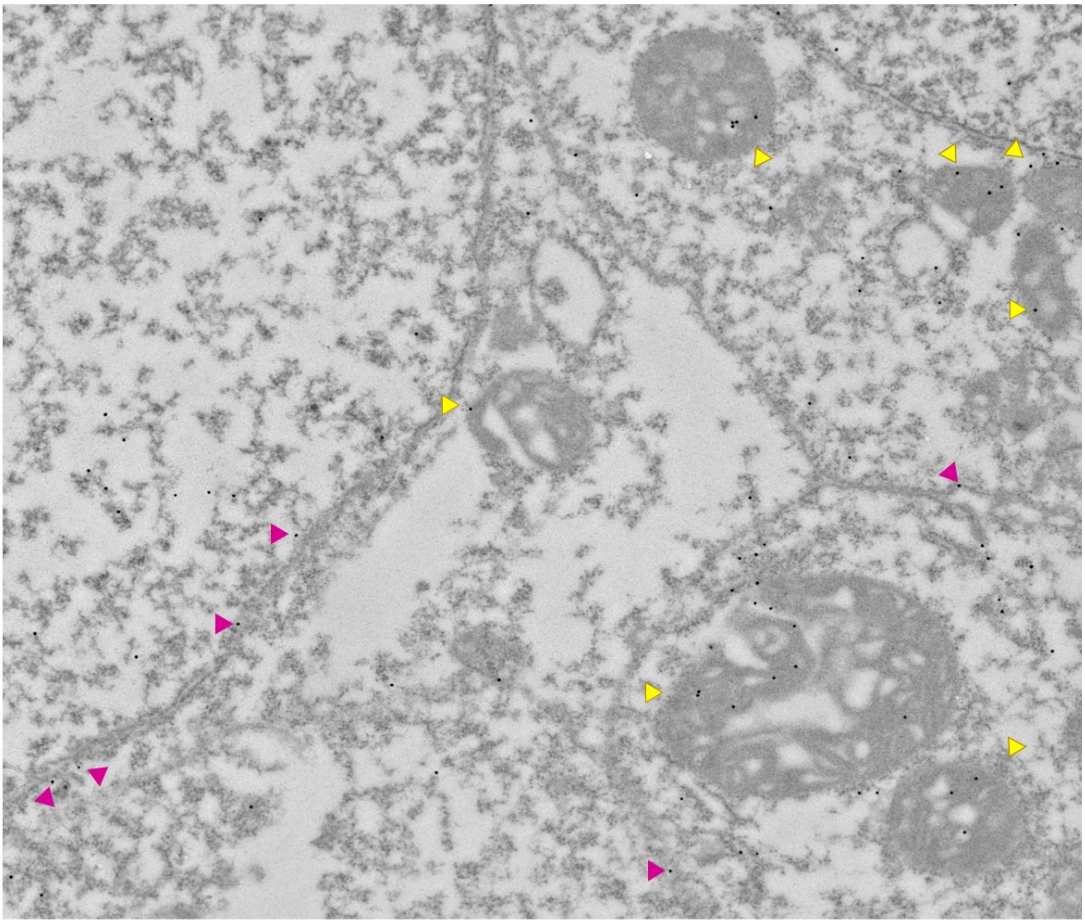

(b)

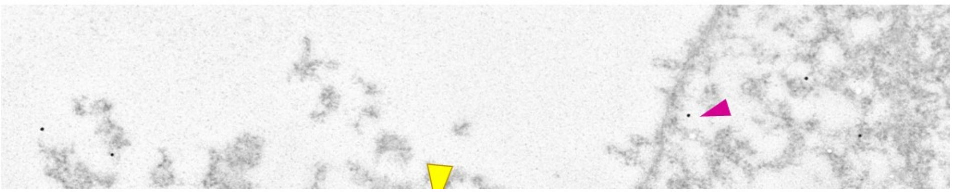

Supplementary Figure S7. 5HT3A subunits on the plasma membrane and mitochondria (a) Transmission electron micrograph image of HEK293T cells transiently transfected with 5HT3A$^{mCherry\text{-}c\text{-}Myc}$ and probed with mouse c-Myc antibody (primary) and 15 nm gold nanoparticle tagged anti-mouse antisera (secondary) to detect 5HT3A$^{mCherry\text{-}c\text{-}Myc}$. 5HT3A$^{mCherry\text{-}c\text{-}Myc}$ subunits present on plasma membrane and mitochondria are indicated by maroon and yellow arrowheads, respectively. Scalebar 1μm. **(b)** 5HT3A$^{mCherry\text{-}c\text{-}Myc}$ subunits on extracellular mitochondria. Transmission electron micrograph images of HEK293T cells transiently transfected with 5HT3A$^{mCherry\text{-}c\text{-}Myc}$ and probed with mouse c-Myc antibody (primary) and 15 nm gold nanoparticle tagged anti-mouse antisera (secondary) to detect 5HT3A$^{mCherry\text{-}c\text{-}Myc}$. Extracellular mitochondria and intracellular mitochondria with 5HT3A$^{mCherry\text{-}c\text{-}Myc}$ are pointed to with yellow and green arrowheads, respectively. 5HT3A$^{mCherry\text{-}c\text{-}Myc}$ on plasma membrane is pointed to with maroon arrowheads. Scalebar 1μm. Antibody details can be found in Supplementary Table 2.

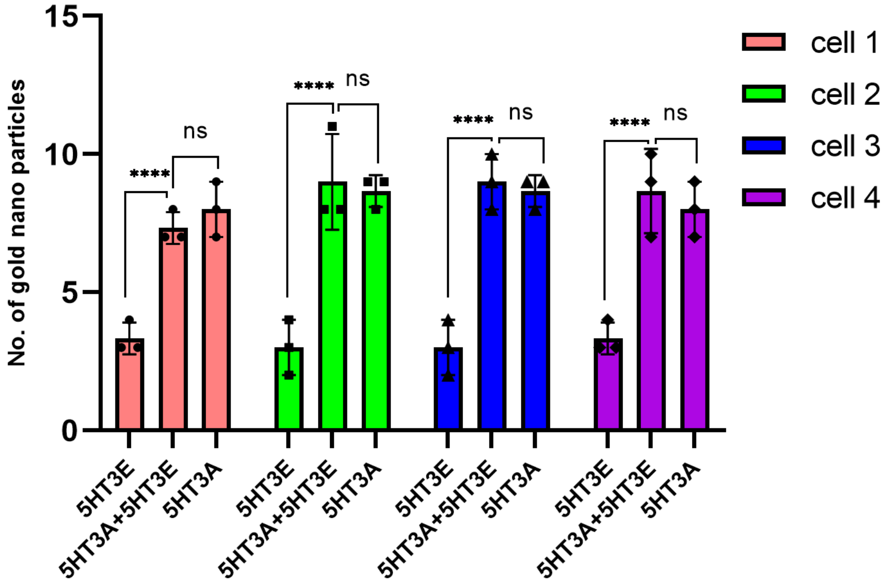

Supplementary Figure S8. Comparison of immunogold particle number on mitochondria of individual cells. Numbers of gold particles detected in cells transfected with 5HT3E$^{mCherry\text{-}HA}$ (6nm gold particles), 5HT3A$^{c\text{-}Myc}$ + 5HT3E$^{mCherry\text{-}HA}$ (6nm + 15nm gold particles), and 5HT3A$^{mCherry\text{-}c\text{-}Myc}$ (15nm gold particles) in the mitochondrial membrane. Data was analyzed using two-way ANOVA followed by Šidák multiple comparison tests (n=4, ****p<0.0001).

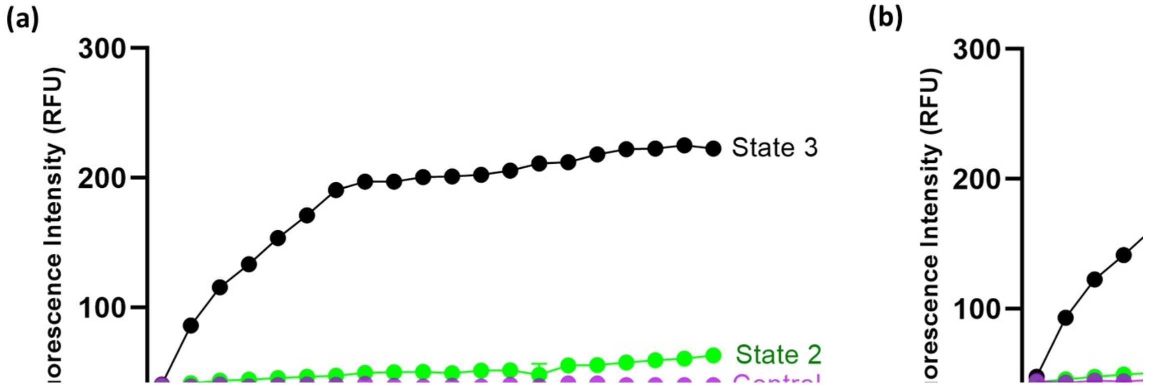

Supplementary Figure S9. Profiles for glutamate malate (12.5 mM) (a) and succinate (25 mM) (b) driven respiration of mitochondria isolated from HEK293T cells. Both basal (state 2) and ADP (1.65 mM)-activated (state 3) profiles are presented. Data points are averages of three replicates.

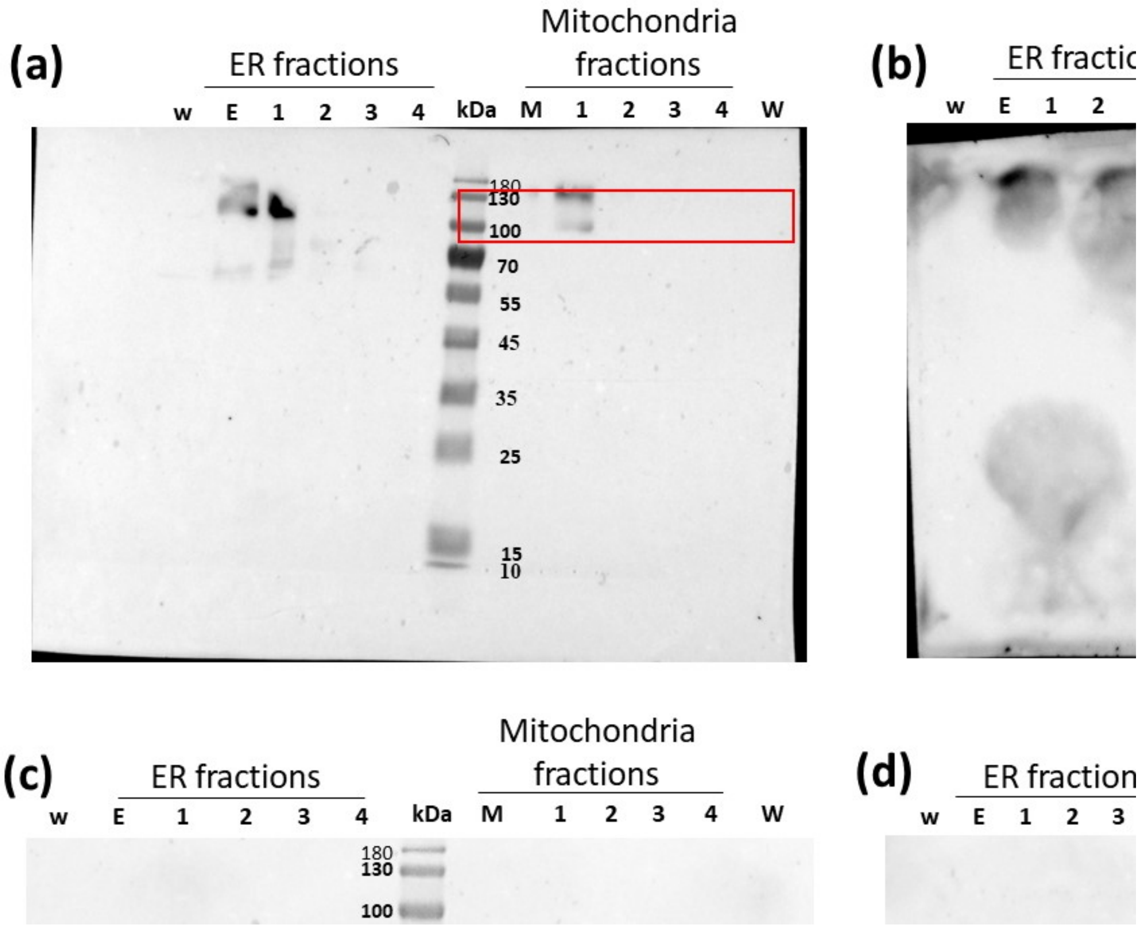

Supplementary Figure S10. Full length immunoblots images corresponding to Figure 2a and Supplementary Figure S6a. Mitochondrial (and endoplasmic reticulum) fractions (1 (10%), 2 (30%), 3 (50%), and 4 (overlaid crude mitochondria)) were isolated from HEK293T cells using an Opti-Prep gradient method (see Figure 2). M is the microsome fraction and W is the whole cell lysate. 20 ug protein was loaded per lane. **(a)** Plasma membrane was probed with Na^+/K^+ ATPase antibody, **(b)** Golgi was probed with Golgin 97 antibody **(c)** Endoplasmic reticulum was probed with disulphide isomerase (PDI) antibody, **(d)** Mitochondria were probed with Translocase of outer mitochondrial membrane22 (TOM22) antibody. The cropped immunoblots in Figure 2a are highlighted by the red box.

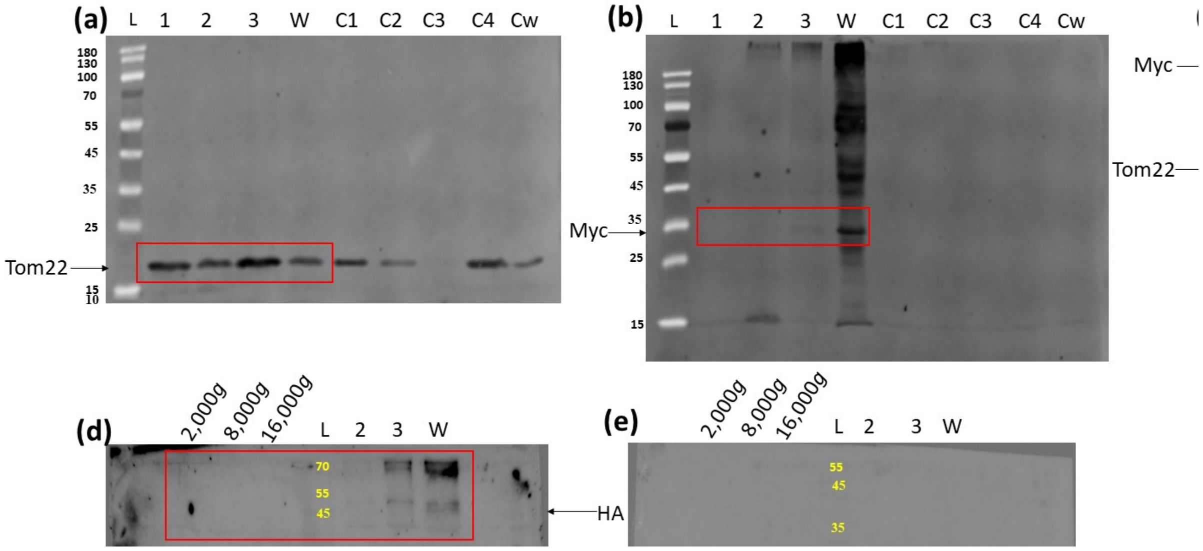

Supplementary Figure S11. Full length immunoblots images corresponding to immunoblot sections shown in Figure 2b, Figure 5a, and Supplementary Figure S4c. (a and b) Full length immunoblots (cropped immunoblots are shown in Figure 2b) of mitochondrial fractions 2 and 3 isolated from cells transfected with 5HT3A$^{mCherry\text{-}c\text{-}Myc}$, probed with TOM22 (a) and c-Myc (b) antibodies. Numbers 1, 2, 3 indicate mitochondrial fractions, W indicates whole cell fraction, C1, C2, C3, C4 and Cw indicate the mitochondria fractions and the whole cell fraction, respectively, from the mock-transfected cells (control). **(c)** Full length immunoblots (cropped immunoblot is shown in Figure 4a) of cell-free mitochondria collected from cell culture media of HEK293T cells transiently transfected with 5HT3A$^{mCherry\text{-}c\text{-}Myc}$ probed with c-Myc antibody (Myc) to detect 5HT3A$^{mCherry\text{-}c\text{-}Myc}$ tagged with c-Myc (bands on the top of the blot) and TOM22 to detect mitochondria membranes (bottom bands). **(d)** and **(e)** mitochondria fractions and extracellular mitochondria were analysed for the presence of 5HT3E$^{mCherry\text{-}HA}$ and (e) Mitochondria were labelled with anti-TOM22, (d) and (e) correspond to immunoblots in Supplementary Figure 5(c). In the blots numbers 2 and 3 represent mitochondria fractions, W represents the whole cell fraction, 2000g, 8000g and 16000g represents the cell-free mitochondria samples collected at the respective centrifugation rates. L denotes ladder. The areas cropped and shown in Figure 2b, Figure 4a, and Supplementary Figure 4c are highlighted by the red boxes.

References

1. Fu, Y.-L.; Zhang, B.; Mu, T.-W. LMAN1 (ERGIC-53) promotes trafficking of neuroreceptors. *Biochem. Biophys. Res. Commun.* **2019**, *511*, 356-362.
2. Shapovalov, G.; Ritaine, A.; Bidaux, G.; Slomianny, C.; Borowiec, A.S.; Gordienko, D.; Bultynck, G.; Skryma, R.; Prevarskaya, N. Organelle membrane derived patches: reshaping classical methods for new targets. *Sci. Rep.* **2017**, *7*, 14082, doi:10.1038/s41598-017-13968-y.
3. Takematsu, H.; Yamamoto, H.; Naito-Matsui, Y.; Fujinawa, R.; Tanaka, K.; Okuno, Y., et al. Quantitative transcriptomic profiling of branching in a glycosphingolipid biosynthetic pathway. *J. Biol. Chem.* **2011**, *286*, 27214-27224.
4. Aristov, A.; Lelandais, B.; Rensen, E.; Zimmer, C. ZOLA-3D allows flexible 3D localization microscopy over an adjustable axial range. *Nat. Commun.* **2018**, *9*, 2409.
5. Avogaro, L.; Querido, E.; Dalachi, M.; Jantsch, M.F.; Chartrand, P.; Cusanelli, E. Live-cell imaging reveals the dynamics and function of single-telomere TERRA molecules in cancer cells. *RNA Biol.* **2018**, *15*, 787-796.
6. Gulbranson, D.R.; Crisman, L.; Lee, M.; Ouyang, Y.; Menasche, B.L.; Demmitt, B.A., et al. AAGAB controls AP2 adaptor assembly in clathrin-mediated endocytosis. *Develop. Cell* **2019**, *50*, 436-446.
7. Larson, S.M.; Truscott, L.C.; Chiou, T.-T.; Patel, A.; Kao, R.; Tu, A., et al. Pre-clinical development of gene modification of haematopoietic stem cells with chimeric antigen receptors for cancer immunotherapy. *Human Vaccines Immunotherap.* **2017**, *13*, 1094-1104.
8. Miyama, A.; Mimura, T.; Noma, H.; Goto, M.; Kamei, Y.; Kondo, A.; Saito, Y.; Okuma, H.; Matsubara, M. Specific IgG for cat allergens in patients with allergic conjunctivitis. *Int. Ophthalmol.* **2015**, *35*, 575-586.
9. Chang, W.; Luo, Q.; Wu, X.; Nan, Y.; Zhao, P.; Zhang, L., et al. OTUB2 exerts tumor-suppressive roles via STAT1-mediated CALML3 activation and increased phosphatidylserine synthesis. *Cell Rep.* **2022**, *41*, 111561, doi:10.1016/j.celrep.2022.111561.
10. Gao, C.; Leng, Y.; Ma, J.; Rooke, V.; Rodriguez-Gonzalez, S.; Ramakrishnan, C.; Deisseroth, K.; Penzo, M.A. Two genetically, anatomically and functionally distinct cell types segregate across anteroposterior axis of paraventricular thalamus. *Nat. Neurosci.* **2020**, *23*, 217-228, doi:10.1038/s41593-019-0572-3.
11. Savojardo, C.; Martelli, P.L.; Fariselli, P.; Casadio, R. TPpred2: improving the prediction of mitochondrial targeting peptide cleavage sites by exploiting sequence motifs. *Bioinformatics* **2014**, *30*, 2973-2974, doi:10.1093/bioinformatics/btu411.
12. Fukasawa, Y.; Tsuji, J.; Fu, S.-C.; Tomii, K.; Horton, P.; Imai, K. MitoFates: improved prediction of mitochondrial targeting sequences and their cleavage sites. *Mol. Cell. Proteomics* **2015**, *14*, 1113-1126, doi:10.1074/mcp.M114.043083.
13. Mól, A.R.; Castro, M.S.; Fontes, W. NetWheels: A web application to create high quality peptide helical wheel and net projections. *bioRxiv* **2018**, 10.1101/416347, doi:10.1101/416347.
14. Al Amir Dache, Z.; Otandault, A.; Tanos, R.; Pastor, B.; Meddeb, R.; Sanchez, C., et al. Blood contains circulating cell-free respiratory competent mitochondria. *FASEB J.* **2020**, *34*, 3616-3630, doi:10.1096/fj.201901917RR.
